# Molecular mechanism of modified KAT2A-mediated histone succinylation in asthma through inhibition of ferroptosis

**DOI:** 10.1016/j.clinsp.2025.100786

**Published:** 2025-09-29

**Authors:** Jian Han, Hui-li Du, Shan-shan Lu, Jun-Feng Li

**Affiliations:** a Department of Respiratory and Critical Care Medicine, Affiliated Hospital of Shandong University of Traditional Chinese Medicine, Shandong Province Hospital of Traditional Chinese Medicine, Jinan, China; b Department of Respiratory and Critical Care Medicine, Feicheng People's Hospital, Taian, China; c Disinfection Supply Center, Guang'anmen Hospital Jinan Hospital (Jinan Municipal Hospital of Traditional Chinese Medicine), Jinan, China; d Pediatrics of Traditional Chinese Medicine, Qingdao Women and Children's Hospital, Qingdao, China

**Keywords:** Asthma, Regulation, Inflammation, Ferroptosis

## Abstract

**Objective:**

KAT2A, as a kind of epigenetic enzyme, has been found to be involved in ferroptosis in recent years, so this research is to explore the role and molecular role of KAT2A-mediated modification of histone succinylation by inhibiting ferroptosis and its involvement in asthma.

**Method:**

An asthma model was established, and the expression of KAT2A, GPX4 and SLC7A11 proteins was analyzed by Western blot and qPCR; Masson staining, TUNEL staining and HE staining were used to observe the pathological changes of lung tissues; The Clone Formation Assay and Cell Counting Kit 8 (CCK-8) were used to assess the cell viability and proliferation; and ELISA was performed for the detection of inflammatory factors; Immune cells were counted with kits. The expression of ferroptosis indicators was evaluated using qPCR and Western blot; ChIP-qPCR was performed to analyze H3K79succ and RNA pol II on the SLC7A11 promoter.

**Results:**

In vitro assays verified that KAT2A regulates asthma through ferroptosis; in vivo assays verified KAT2A-mediated IL-13 succinylation modification and its effect on asthma; KAT2A controls ferroptosis via SLC7A11 and subsequently regulates asthma; Erastin significantly increased the levels of Fe2+, lipid ROS, SOD, Iron, and MDA, and decreased the expression of GPX4 and SLC7A11 and reduced inflammatory response.

**Conclusions:**

The authors verified that KAT2A can reduce the inflammatory response caused by asthma, and further clarified that KAT2A-mediated histone succinylation modification participates in the occurrence of asthma by inhibiting ferroptosis, which may become a potential target for asthma treatment.

## Highlights

• KAT2A, epigenetic enzyme has been found to be involved in iron death.
• Asthma model shown the expression of KAT2A, GPX4 and SLC7A11.
• Erastin significantly increased the levels of Fe2+, lipid ROS, SOD, Iron, and MDA.
• Decreased the expression of GPX4 and SLC7A11 and reduced inflammatory response.
• KAT2A-mediated histone succinylation by inhibiting iron death provides therapeutic.

## Introduction

Asthma, a common chronic inflammatory airway disease characterized by airway inflammation, remodeling, and hyperresponsiveness, significantly impacts the quality of life and health status of a large global population^1^ According to the World Health Organization, approximately 235 million people worldwide suffer from asthma, with varying prevalence rates across different regions. In some high-incidence areas such as Australia, the prevalence rate can reach up to 21 %^2^ In China, the number of asthma patients has exceeded 45 million, with a prevalence rate of approximately 4.2 %^3^ Due to factors like environmental pollution, smoking, allergies, and population aging, the incidence and mortality rates of asthma are gradually increasing, posing a heavy medical burden on individuals and society^4^ Therefore, it is urgent to deeply explore the pathophysiology of asthma and identify effective therapeutic targets.

The pathophysiology of asthma is highly complex, involving multiple cellular and molecular mechanisms. Recently, the role of ferroptosis, a novel cell death mode, in the pathogenesis of asthma has garnered increasing attention^5^^,^^6^ Ferroptosis is a form of cell death triggered by lipid peroxidation, where iron accumulation leads to increased generation of lipid Reactive Oxygen Species (ROS), ultimately damaging the cell membrane structure^7^ In the airway epithelial cells of asthma patients, key components of the ferroptosis defense system, such as cystine/glutamate antiporter SLC7A11 and Glutathione Peroxidase 4 (GPX4), play crucial roles. SLC7A11 mediates cystine uptake to maintain GPX4 activity, thereby neutralizing lipid peroxides and inhibiting ferroptosis; however, when SLC7A11 and GPX4 functions are impaired, ferroptosis can propagate in airway epithelial cells. Studies have found reduced SLC7A11/GPX4 expression and elevated levels of the lipid peroxidation marker 4-HNE in bronchial biopsy samples from patients with severe asthma, indicating a close association between decreased anti-ferroptosis capacity and asthma severity^8^^,^^9^

Lysine Acetyltransferase 2A (KAT2A), also known as General Control Non-repressible protein 5 (GCN5), is an important epigenetic enzyme belonging to the GNAT family. It alters DNA structure by transferring acetyl groups, enhancing the transcription level of specific DNA, and regulating various biological processes^10^ Recent studies have revealed that KAT2A plays a key role in regulating cellular redox homeostasis. Besides its classical function in histone acetylation, KAT2A can directly modify metabolic enzymes in the glutathione synthesis pathway through succinylation^11^ Mounting evidence suggests that KAT2A may participate in the development of various diseases by regulating ferroptosis, although its specific mechanistic role in asthma remains elusive.

Currently, it is hypothesized that KAT2A-mediated succinylation may directly inhibit ferroptosis via the SLC7A11/GPX4 axis. As an enzyme capable of regulating gene transcription, KAT2A may modify the promoter region of the SLC7A11 gene, influencing its transcription process. Specifically, KAT2A could catalyze histone succinylation, altering chromatin structure and facilitating the binding of transcription factors to the SLC7A11 gene promoter region, thereby promoting SLC7A11 expression. Elevated SLC7A11 expression enables increased cystine uptake, maintaining intracellular Glutathione (GSH) levels and providing sufficient substrates for GPX4, enhancing its activity and effectively inhibiting lipid peroxidation and ferroptosis. However, this hypothesis requires further experimental validation.

To clarify the role and mechanism of KAT2A in asthma, a series of experiments was conducted in this study. Regarding the detection of inflammatory factors, given the involvement of multiple cytokines in the inflammatory response during asthma pathogenesis, and based on numerous previous studies demonstrating the key roles of Interleukin-4 (IL-4), Interleukin-13 (IL-13), and Tumor Necrosis Factor-alpha (TNF-α) in asthma inflammation, these cytokines were selected for investigation^12^^,^^13^ IL-4 promotes IgE production by B-cells, playing a crucial role in allergic reactions and airway inflammation in asthma; IL-13 induces high secretion of airway mucus, promotes airway smooth muscle contraction and remodeling; TNF-α activates inflammatory cells, facilitating the release of inflammatory mediators and exacerbating airway inflammation.

The authors validate the protective effect of KAT2A through Erastin. As an inducer of ferroptosis, if KAT2A indeed exerts a protective effect on asthma by inhibiting ferroptosis, the introduction of Erastin to induce ferroptosis should weaken or reverse this protective effect^14^ Through these experimental results, the authors aim to further elucidate the specific mechanism by which KAT2A-mediated histone succinylation modifies and inhibits ferroptosis, thereby participating in the asthmatic process. This will provide new potential targets and a theoretical basis for asthma treatment.

## Materials and methods

### Cell culture and cell transfection and treatment

HBE cells were purchased from the Shanghai Cell Bank of the Chinese Academy of Sciences (Shanghai, China) and maintained in a 37 °C incubator containing fetal bovine serum (10 % of FBS; BI, China) and 1 % streptomycin/penicillin (Sigma, USA) filled with 5 % CO_2_. The siRNA targeting the KAT2A gene was ordered from RiboBio (Guangzhou, China). siRNA was transfected into cells with PEI reagent and Lipofectamine RNAiMAX and kept for 48 hrs. After that, cells were treated with cycloheximide (CHX, 200 ng/mL) for 24 h to inhibit protein synthesis. pCDNA3.1 for the construction of the KT2A overexpression vector was purchased from Thermo Fisher Scientific Inc. (USA).

### Construction of an OVA-induced asthma mouse model

A total of 32 male C57BL/6 J mice (6‒8 weeks) were procured from Beijing Vital River Laboratory Animal Technology Co., Ltd. Except for 10 controls, the other mice were challenged by intraperitoneal injection of 0.2 mL of saline solution containing 20 μg of OVA and 2 mg of aluminum hydroxide on day 0 and day 7, from days 14 to 16, mice were inhaled with 3 % OVA solution by ultrasonic nebulizer for 30 min each time. Mice were challenged with 3 % OVA on days 21 to 23. All animal studies follow the ARRIVE guidelines and the ethics batch number is MDL2023-10-19-01.

### Quantitative real-time PCR

Total RNA was extracted from cells using Trizol reagent (Thermo, USA) and reverse transcribed to cDNA by using the First-strand synthesis kit (Transgene, China). RNA level was measured using a SYBR Green Supermix (Transgene) and detected using the Real-Time PCR Detection System (Bio-Rad, USA). The β-Actin level was set as an internal reference for normalization.

### Western blot assay

Total proteins from the mouse HBE cells were extracted by using a lysis buffer and then were separated using SDS-PAGE. After that the protein in the gel was transferred into membranes. After blocking in 5 % non-fat milk, protein bands were probed with antibodies for caspase3, LC3, p62, pAKT, AKT, p-mTOR, mTOR, and β-actin overnight at 4 °C. After that, the membrane was immersed in secondary antibody conjugated HRP and then wash the membrane with washing buffer. Finally, the ECL reagent was added to see the protein bands. All antibodies were procured from Abcam (USA).

### Chromatin immunoprecipitation (ChIP) assay

The ChIP Assay Kit (P2078, Beyotime) was applied for ChIP detection. Chromatin was cross-linked in 1 % formaldehyde at 37 °C for 10 min, sonicated to a size of 200‒1000 bp, and then incubated with anti-SLC7A11/H3K79succ/RNA pol II for 8 h at 4 °C, followed by incubation with Protein A + G Agarose for 1 h at 4 °C. After elution of the protein/DNA complex. The DNA was de-crosslinked. Immunoprecipitated DNA was analyzed by RT-qPCR.

RNA polymerase II: abcam, ab300575

SLC7A11: abcam, ab302919

H3K79succ: PTM BIO, PTM-412

### CCK-8 assay

The cells in sh-NC and sh-METTL3 groups were treated with different concentration gradients of Cisplatin, and a CCK-8 assay was performed 24 h later. The cells were incubated at 37 °C for 4 h in a 5 % CO_2_ incubator, the supernatant was discarded, and the cells were incubated with DMSO for 10 min, the absorbance at 450 nm was measured on an enzyme counter, and the cell activity was calculated.

### Histochemical staining

Immunohistochemical staining, xylene I dewaxing for 10 min. Xylene II dewaxing for 10 min. Gradient alcohol hydration: 100 % alcohol, 95 % alcohol, 80 % alcohol, 70 % alcohol. Soak in each gradient of alcohol for 5 min. The sections were rinsed in running water for 10 min. and in PBS 3 times for 5 min each. Place sections in prepared sodium citrate solution and thaw in a microwave oven for 20 min. Cool at room temperature. Rinse 3 times with PBS for 5 min each time. Add 0.3 % hydrogen peroxide solution dropwise on top of the tissue and incubate at 4 °C for 15 min. The tissue was rinsed 3 times with phosphate buffer solution at 5-minute intervals. Incubate with 10 % goat serum for 30 min at 37 °C. Dilute USP37 antibody with PBS, add an appropriate amount of USP37 antibody dilution to each tissue, and incubate at 4 °C overnight. Run 3 times in PBS, each time 5 min apart. An appropriate amount of biotin secondary antibody was added to each tissue dropwise and incubated at 4 °C for 30 min. and washed with PBS 3 times, each time with an interval of 5 min. Horseradish peroxidase was added dropwise to the tissue and incubated at 4 °C for 30 min. and washed with PBS 3 times, each time with an interval of 5 min. The color was developed by adding DAB colorant and rinsing in PBS for 10 min. Sections were placed in hematoxylin for 2 min, soaked in warm water and rinsed in PBS for 10 min. Gradient alcohol 70 % alcohol, 80 % alcohol, 95 % alcohol, 100 % alcohol. Each was soaked for 5 min.

### Masson staining

Masson staining also followed the kit guidelines, and was performed sequentially with ferric hematoxylin staining for 5 min, water washing, 1 % hydrochloric acid ethanol staining for 1 s, water washing, Li Chun red staining for 10 min, phosphomolybdic acid staining for 1∼5 min, and toluidine blue water washing, and finally washed with 1 % glacial acetic acid for 1 min and dehydrated and sealed, and was observed under a microscope and photographed with pathological pictures (with a magnification of 200 ×), and the percentage of the stained area to the total area was calculated with Image J fiber to calculate the percentage of stained area to total area.

### TUNEL staining

Cells were seeded on the bottom of the transwell 6-well plates with cell crawlers to construct a co-culture model. After intervention, TUNEL staining was performed: discard the original culture medium, add fixative and incubate at room temperature for 20 min, add membrane-breaking solution and incubate for 5 min, add buffer and incubate for 10 min, take an appropriate amount of TDT enzyme and incubate at 37 °C for 1 h. Add DAPI staining solution and incubate for 10 min, avoiding light, and then seal the plate. Observe and collect images under a fluorescence microscope.

### MDA assay for lipid peroxidation levels

Cells were collected and operated according to the kit instructions. Each well absorbance was measured using a multifunctional enzyme marker, and the MDA level was calculated.

Fe2+ content assay

Cells were collected and operated according to the kit instructions. Determine the absorbance value of each well using a multifunctional enzyme marker and calculate the Fe2+ content according to the standard curve.

ROS level

The procedure was performed according to the instructions of the Reactive Oxygen Kit. Cells were collected and resuspended, DCFH-DA was added, mixed, and incubated at 37 °C for 20 min. Fluorescence intensity was detected by flow cytometry and analyzed by FlowJo software.

### ELISA

Blank, standard, and sample groups were tested with the ELISA assay. Briefly, 50 µL of standard samples was added to the enzyme-labeled coating plate. For samples to be tested, 40 µL of sample dilution was added to the sample group, and then 10 µL of the samples to be tested was added, and then gently shaken well. Incubate at 37 °C for 30 min and then wash with detergent, and repeat 5 times to remove the tissue solution. Add developer A and developer B to each group, shake gently, and keep at 37 °C for 10 min in the dark, return the blank group to 0, and measure the absorbance at 450 nm (A) of each group in turn. Three samples were measured in each group, and each sample was tested three times.

### Clone formation assay

Cell suspensions were prepared, and each group of cells was inoculated in 6-well plates with 500∼1000 cells per well, respectively, and 2 mL of complete medium was added, and cultured for about 1 week until clone spheres visible to the naked eye were produced. After methanol fixation for 30 min, the cells were stained with Giemsa's stain, rinsed and air-dried, photographed and the number of clone formation was calculated for each group.

### Statistical analysis

The data analysis was conducted using GraphPad Prism 6.0 software (San Diego, CA, USA) and presented as mean ± S.D. Each experiment had three replicates (*n* = 3). For comparisons among multiple groups, the authors utilized one-way analysis of variance (ANOVA) followed by Tukey's multiple comparison posttest to assess statistical significance. Additionally, Student's unpaired *t*-test was employed to compare differences between two groups. Statistical significance was defined at *p* < 0.05.

## Results

### In vitro assay verifies that KAT2A regulates asthma through ferroptosis

**Fig. 1:**
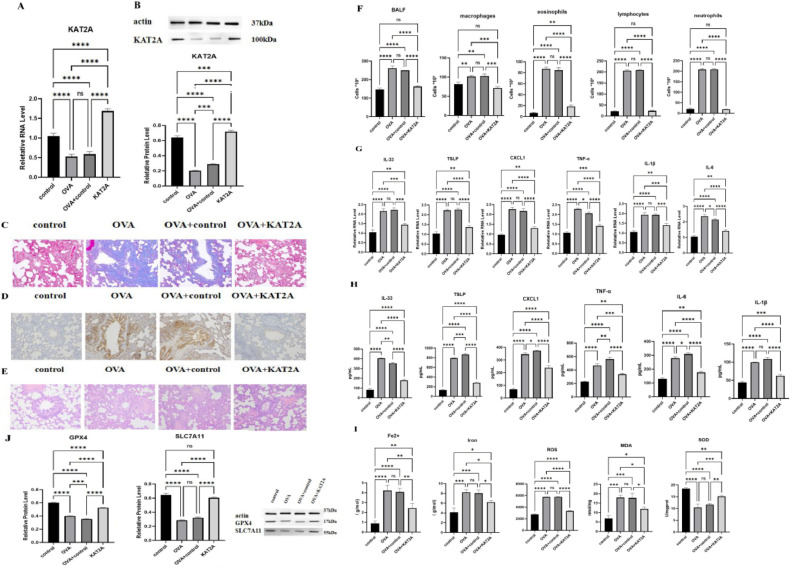
In vivo assay verifies that KAT2A regulates asthma through ferroptosis (A‒J). qPCR analysis of KAT2A in C57BL/6 J (A), WB analysis of KAT2A in C57BL/6 J (B), Masson staining of C57BL/6 J (C), TUNEL staining of C57BL/6 J (D), H&E staining of C57BL/6 J (E), Immuno Cytometrics including the cells level of BALF, macrophages, eosinophols, lymphocytes, neutrophils in C57BL/6 J (F), qPCR analysis of inflammatory factors in C57BL/6 J (G), ELISA analysis of inflammatory factors in C57BL/6 J (H), Fe2+, Iron, lipid ROS, MDA, SOD analysis (I), WB analysis of GPX4 and SLC7A11 in C57BL/6 J (J). **** *p* > 0.0001, *** *p* > 0.001, ** *p* < 0.01, * *p* < 0.05, ns: not significant.

The authors constructed the OVA asthma model, and through qPCR experiment and protein immunoblotting experiment, it was found that the protein in the OVA model group had low expression, and the protein in the KAT2A group had high expression and was significantly higher than that in the blank control group (*p* < 0.01) (Fig. 1A and B). Masson staining results of the lung tissue showed that the blank group bronchial tissue structure is normal, no airway wall and smooth muscle thickening, no obvious inflammatory cell infiltration (Fig. 1C); OVA asthma model group can be seen around the inflammatory cell infiltration is obvious, the blue collagen deposition around the tracheal blood vessels further aggravated, epithelial cell fibrosis; OVA + control group have different degrees of pathology changes. In the OVA + control group, the pathological changes were reduced to different degrees, with a small amount of inflammatory cell infiltration around the airways, reduced thickness of the basement membrane and smooth muscle, and reduced collagen deposition; in the OVA+KAT2A group, the tissue results returned to normal, with no obvious inflammatory cell infiltration, and TUNEL staining showed that the nuclei of the cells in the control group were blue, while the nuclei of apoptotic cells were stained in brownish-yellow in the OVA asthma model group, with a significant increase in the number of positive cells; the number of positive cells was significantly reduced in the OVA+KAT2A group (Fig. 1D). HE staining showed that in the blank group, the bronchial mucosa was smooth and intact, the lumen was smooth, the alveolar structure was intact, there was no thickening of the smooth muscle layer and the wall, and there was no obvious inflammatory cell infiltration; in the OVA model group, a large number of inflammatory cell infiltration was seen in the lung tissues, with disorganized airway epithelial structure, edema of mucous membranes, obvious narrowing of lumen, and a large number of inflammatory cells exuding from the peripheral part of the bronchial tubes; in the OVA+KAT2A group, the nuclei of the bronchial mucosa were blue, and the nuclei of apoptotic cells were stained in brownish-yellow, with a significantly greater number of positive cells. KAT2A group bronchial mucosal congestion and edema was reduced, inflammatory cells were reduced, the basement membrane was mildly hyperplastic and hypertrophic, and epithelial cell shedding was reduced (Fig. 1E). In order to investigate the effect of KAT2A on inflammation, the authors counted macrophages, eosinophils, lymphocytes, and neutrophil cells, and found that the number of BALF, macrophages, eosinophils, lymphocytes, and neutrophils cells in the OVA asthma model group was significantly higher than that in the blank group (*p* < 0.01), and with the addition of KAT2A, the number of these cells decreased, especially the number of cells of eosinophils, lymphocytes, and neutrophils decreased significantly (*p* < 0.01) (Fig. 1F). Next, inflammatory factors were detected by qPCR and verified by ELISA, and it was found that inflammatory factors were significantly highly expressed in the OVA asthma model group (*p* < 0.01), and with the addition of KAT2A, the expression of inflammatory factors was significantly reduced (*p* < 0.01) (Fig. 1G and H). Changes in the levels of Fe2+, Iron, lipid ROS, MDA, and SOD in lung tissues were observed using biochemical assays, and the results showed that the expression was significantly higher in the model group compared with the control group (*p* < 0.01), and significantly lower with the addition of KAT2A (*p* < 0.01) (Fig. 1I). Detection of GPX4 and SLC7A11 levels in lung tissues using Western botting showed a significant decrease (*p* < 0.01) in the model group, and again KAT2A reversed this result (Fig. 1J).

### In vitro assay to validate KAT2A-mediated histone succinylation modification and its effect on asthma

**Fig. 2:**
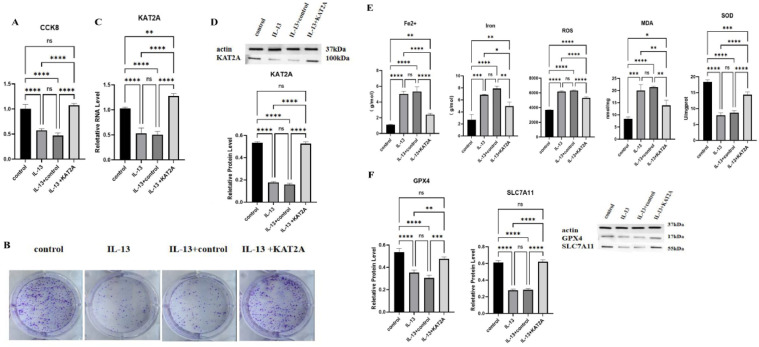
In vitro assay to validate KAT2A-mediated histone succinylation modification and its effect on asthma (A‒F) CCK-8 analysis of cell proliferation (A), Clone formation analysis of cell proliferation (B), qPCR analysis of KAT2A in HBE cells (C), WB analysis of KAT2A in HBE cells (D), Fe2+, Iron, lipid ROS, MDA, SOD analysis in HBE cells (E), WB analysis of GPX4 and SLC7A11 in HBE cells (F). **** *p* > 0.0001, *** *p* > 0.001, ** *p* < 0.01, * *p* < 0.05, ns: not significant. Each experiment had three replicates (*n* = 3), the data analysis was conducted using GraphPad Prism 6.0 software (San Diego, CA, USA) and presented as mean ± S.D. and presented as mean ± S.D.

The viability of HBE cells was detected by CCK-8 and cell cloning assay, which was firstly categorized into four groups: control, IL-13, IL-13+control, and IL-13 + KAT2A, and it was found that IL-13 caused a significant decrease in cell viability, and KAT2A reversed this result (Fig. 2A and B). KAT2A was then analyzed by qPCR assay (Fig. 2C) and protein immunoblotting assay (Fig. 2D), and as with the previous results, protein expression was significantly reduced in the IL-13 group (*p* < 0.01) and increased in the IL-13 + KAT2A group (*p* < 0.01). Then the authors used biochemical assays to observe the changes in the levels of Fe2+, Iron, lipid ROS, MDA, and SOD, and found that IL-13 significantly elevated the levels of Fe2+, Iron, lipid ROS, MDA, and SOD (*p* < 0.01), and KAT2A reversed this result (Fig. 2E). Finally, GPX4 and SLC7A11 levels were detected using Western botting, and it was found that the levels of GPX4 and SLC7A11 were significantly decreased in the IL-13 group (*p* < 0.01), and significantly increased in the IL-13 + KAT2A group (*p* < 0.01) (Fig. 2F).

### KAT2A regulates asthma through SLC7A11

**Fig. 3:**
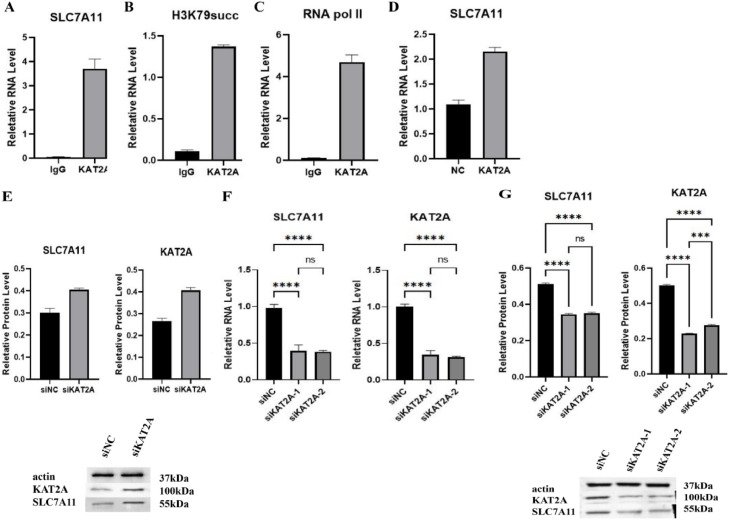
KAT2A regulates asthma through SLC7A11 (A-G) ChIP-qPCR analysis of SLC7A11 (A), ChIP-qPCR analysis of H3K79succ on the promoter of SLC7A11 (B), ChIP-qPCR analysis of the promoter of SLC7A11 on RNA pol II (C), qPCR analysis of SLC7A11 (D and F), WB analysis of KAT2A and SLC7A11 (E and G). **** *p* > 0.0001, *** *p* > 0.001, ** *p* < 0.01, * *p* < 0.05, ns: not significant. Each experiment had three replicates (*n* = 3), the data analysis was conducted using GraphPad Prism 6.0 software (San Diego, CA, USA) and presented as mean ± S.D. and presented as mean ± S.D.

SLC7A11, as an important component of the Xc-system, a classical pathway of ferroptosis, can effectively regulate the process of cellular ferroptosis. In order to study how KAT2A regulates asthma through SLC7A11, the authors analyzed SLC7A11 by ChIP-qPCR, and found that SLC7A11 was significantly increased in the KAT2A group (*p* < 0.01) (Fig. 3A), and then analyzed the expression of H3K79succ and RNA pol II on the promoter of SLC7A11, as shown in the figure, KAT2A significantly increased H3K79succ and RNA pol II expression (*p* < 0.01) (Fig. 3B and C). To further investigate the relationship between KAT2A and SLC7A11, SLC7A11 was analyzed by qPCR and immunoblotting assay, which showed that SLC7A11 expression was significantly increased in the KAT2A group (*p* < 0.01) (Fig. 3D and E). So the authors transfected with siKAT2A-1 and siKAT2A-2 and analyzed KAT2A and SLC7A11 by qPCR and WB, and the results showed that silencing KAT2A significantly decreased the expression of SLC7A11 (*p* < 0.01) (Fig. 3F and G). This suggests that KAT2A controls ferroptosis and subsequently regulates asthma through SLC7A11.

### KAT2A-mediated histone succinylation modification is involved in asthma by inhibiting ferroptosis

**Fig. 4:**
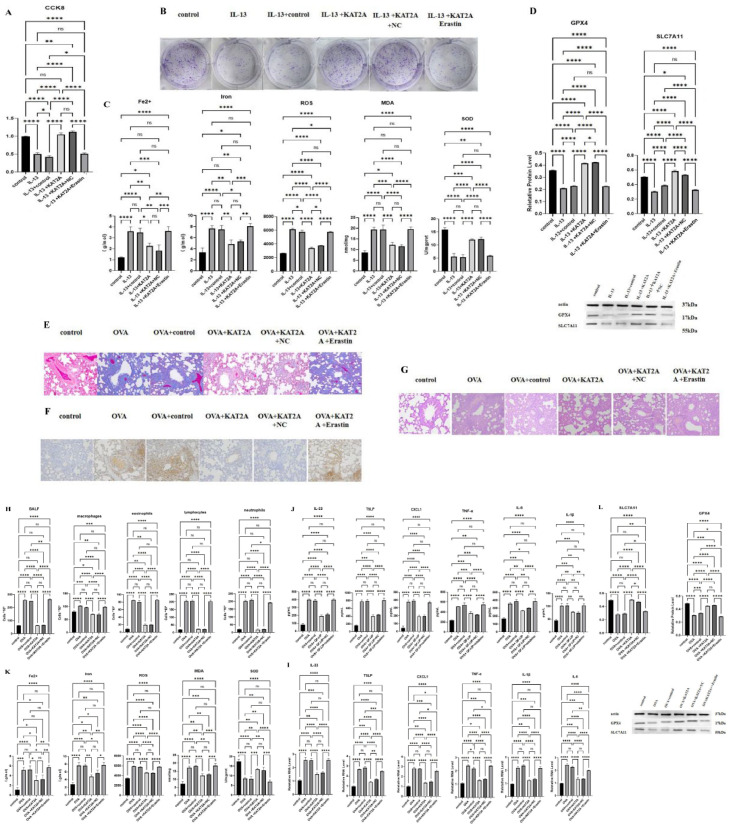
KAT2A-mediated histone succinylation modification is involved in asthma by inhibiting ferroptosis (A‒L) CCK-8 analysis of cell proliferation (A), Clone formation analysis of cell proliferation (B), Fe2+, Iron, lipid ROS, MDA, and SOD analysis (C and K), WB analysis of GPX4 and SLC7A11 (D and L), Masson staining (E), TUNEL staining (F), H&E staining (G), Immunocytometrics (H), qPCR analysis of inflammatory factors (I), ELISA analysis of inflammatory factors (J).

To further validate that KAT2A regulates asthma by inhibiting ferroptosis, the authors added the ferroptosis inducer Erastin and observed whether the protective effect of KAT2A would be reversed. The authors investigated the effect of Erastin on HBE cell viability,then analyzed cell viability using CCK-8 and cell clone formation assay, and the results showed that KAT2A significantly increased cell viability (*p* < 0.01), which was directly reversed by Erastin (Fig. 4A and B). The authors then analyzed Fe2+, Iron, lipid ROS, MDA, and SOD, and found that Erastin significantly increased the levels of Fe2+, Iron, lipid ROS, MDA, and SOD (*p* < 0.01) (Fig. 4C). WB results showed that Erastin significantly decreased the expression of GPX4 and SLC7A11 (*p* < 0.01) (Fig. 4D). The authors stained the lung tissues of OVA asthma model, Masson staining results showed that Erastin caused significant inflammatory cell infiltration in lung tissues, further aggravation of blue collagen deposition around the tracheal blood vessels, and fibrosis of epithelial cells (Fig. 4E). TUNEL staining results showed that Erastin stained nuclei of apoptotic cells brownish-yellow color, and the number of positive cells was significantly increased (Fig. 4F). HE staining results showed that the results of HE staining showed that the lung tissue of OVA+KAT2A+Erastin group was infiltrated with a large number of inflammatory cells, the airway epithelial structure was disorganized, the mucous membrane was edematous, the lumen was obviously narrowed, and there was a large number of inflammatory cells exuding from the peribronchial area (Fig. 4G). In order to investigate whether Erastin could improve the inflammatory response in asthma, the authors counted macrophages, eosinophils, lymphocytes, and neutrophilic leukocytes, and found that Erastin significantly increased the number of immune cells (*p* < 0.01) (Fig. 4H). Next, inflammatory factors were detected by qPCR and verified by ELISA, and with the addition of KAT2A, the expression of inflammatory factors was significantly reduced (*p* < 0.01), a result that was directly reversed after the addition of Erastin (Fig. 4I and J). Finally, the authors used biochemical assays to observe the changes in the levels of Fe2+, Iron, lipid ROS, MDA, and SOD in lung tissues, and found that Erastin significantly increased their levels while significantly reducing the expression of GPX4 and SLC7A11 (*p* < 0.01) (Fig. 4K and L).

## Discussion

Asthma is a chronic inflammatory airway disease characterized by recurrent wheezing, shortness of breath, chest tightness, or coughing^15^ Due to urbanization and changes in lifestyle, the prevalence of asthma has been increasing yearly. It not only severely affects patients' quality of life but also consumes significant healthcare resources, exacerbating the socioeconomic burden. Therefore, effective prevention and treatment of asthma remain crucial clinical tasks and challenges. Previous studies have found that ferroptosis is involved in the pathogenesis of asthma, and the acetylation modification of KAT2A is associated with the regulation of inflammation, oxidative stress, and lipid metabolism^16^^,^^17^ In this study, the authors delved into the role and mechanism of KAT2A-mediated histone succinylation in asthma progression by inhibiting ferroptosis.

In exploring asthma treatment mechanisms, animal models are often used to investigate the pathophysiology and therapeutic approaches of asthma^18^ This study constructed an OVA-induced asthma model to verify the role of KAT2A in regulating asthma through ferroptosis. Asthma, a complex and heterogeneous disease arising from gene-environment interactions, is primarily characterized by chronic airway inflammation and airway remodeling. Airway remodeling, the main cause of progressive lung function decline and irreversible airflow limitation in asthma patients, involves pathological changes such as subepithelial fibrosis, airway smooth muscle hypertrophy and hyperplasia, collagen deposition, and angiogenesis^19^^,^^20^ In the present study, the authors observed that KAT2A reduced inflammatory cell infiltration, decreased basement membrane and smooth muscle thickness, and diminished collagen deposition. Conversely, Erastin, a small-molecule antitumor drug and the first discovered ferroptosis inducer, led to significant inflammatory cell infiltration in lung tissue, aggravated blue collagen deposition around airway blood vessels, and induced epithelial cell fibrosis^14^^,^^21^ Asthma is a chronic inflammatory respiratory disease mediated by various inflammatory cells and components (such as BALF, macrophages, eosinophils, lymphocytes, and neutrophils), often accompanied by airway inflammation, airway hyperreactivity, and reversible airflow obstruction^18^ This analysis revealed that KAT2A decreased the number of these cells, while Erastin reversed this effect. Additionally, Interleukin-13 (IL-13), a pleiotropic regulatory factor specific to T-helper type 2 cells, plays a pivotal role in the pathogenesis of asthma by widely participating in inflammatory and immune diseases. Through in vivo experiments, the authors verified that KAT2A mediates the succinylation of IL-13 and is involved in asthma development.

Regarding how KAT2A-mediated succinylation directly regulates H3K79succ on the SLC7A11 promoter, although current research has shown that KAT2A significantly increases the expression of H3K79succ and RNA polymerase II on the SLC7A11 promoter, the specific molecular mechanism remains incompletely understood^22^^,^^23^ It is hypothesized that KAT2A, as a histone-modifying enzyme, may directly bind to the SLC7A11 promoter region, transferring a succinyl group to the lysine 79 site (H3K79) of histone H3. This modification could alter the structure and function of chromatin, shifting it from a tight transcriptionally repressed state to an open and transcriptionally active state. Consequently, this promotes the binding of RNA polymerase II to the promoter, enhancing SLC7A11 transcription.

The antioxidant system is a crucial defensive mechanism during ferroptosis. The System Xc-, a cystine/glutamate antiporter on the cell membrane, consists of SLC7A11 and SLC3A2 connected by a disulfide bond to form a heterodimer^24^ SLC7A11, a vital component of the ferroptosis classic pathway Xc-system, effectively regulates the process of ferroptosis. Within the System Xc-, Glutathione Peroxidase 4 (GPX4) inhibits the hydroperoxidation of lipids such as phospholipids and cholesterol, and collaborates with GSH to reduce the accumulation of lipid hydroperoxides, thereby suppressing ferroptosis^25^ Studies have indicated that low maternal blood selenium concentrations can lead to decreased GPX4 activity in fetal airway epithelial cells, impairing antioxidant defense capabilities and contributing to epithelial damage involved in asthma development^26^ To investigate how KAT2A regulates asthma through SLC7A11, the authors employed ChIP-qPCR analysis and found that KAT2A significantly increased the expression of H3K79succ and RNA polymerase II on the SLC7A11 promoter. This suggests that KAT2A controls ferroptosis through modulation of SLC7A11, thereby regulating asthma.

Ferroptosis regulation occurs through multiple pathways, and Damage-Associated Molecular Patterns (DAMPs) represent one of the significant routes for ferroptosis to exert its biological effects. DAMPs are endogenous danger signals released by the body upon injury, including High-Mobility Group protein B1 (HMGB1), Interleukin-1β (IL-1β), Tumor Necrosis Factor α (TNF-α), Vascular Endothelial Growth Factor (VEGF), and Interleukin-33 (IL-33)^27^ The examination of these inflammatory factors revealed that KAT2A significantly reduced their levels, while Erastin reversed this outcome. Furthermore, ferroptosis is a form of cell death characterized by the destruction of the cell membrane structure due to lipid peroxidation. Its primary features include mitochondrial morphological shrinkage, elevated ROS levels, GSH depletion, abnormal iron metabolism, and the accumulation of lipid peroxidation products^7^ The analysis of indicators such as Fe2+, iron ions, lipid ROS, MDA, and SOD demonstrated that KAT2A lowered their levels, whereas Erastin significantly elevated these markers and concomitantly reduced the expression of GPX4 and SLC7A11.

From a clinical perspective, current asthma treatments primarily focus on anti-inflammatory and bronchodilator approaches^28^ However, the therapeutic effectiveness remains unsatisfactory for some patients, emphasizing the importance of identifying new therapeutic targets. The present study reveals that KAT2A-mediated histone succinylation is involved in asthma progression by inhibiting ferroptosis, offering a potential new target for asthma treatment. Compared to existing anti-IL-13 biologics, which alleviate asthma inflammation by blocking the IL-13 signaling pathway but can only intervene in a single cytokine and may cause drug resistance or adverse reactions in some patients, targeting KAT2A could regulate asthma pathogenesis at a more upstream epigenetic level. This approach not only modulates IL-13-related inflammatory responses but also impacts multiple pathological aspects of asthma, such as reducing airway epithelial cell damage and improving airway remodeling, by inhibiting ferroptosis^29^^,^^30^ Additionally, as an enzyme, KAT2A has the potential to be targeted by small-molecule inhibitors or activators. Compared to biologics, small-molecule drugs offer better stability, lower production costs, and more convenient administration routes. However, it's important to note that this discovery is still in the basic research stage, and further studies are needed to translate it into clinical applications. This includes validating KAT2A's role in larger clinical samples, developing specific drugs targeting KAT2A, and evaluating their safety and effectiveness. Nonetheless, this study opens up new avenues for asthma treatment with potential clinical translational value.

In conclusion, this study provides initial insights into the mechanistic role of KAT2A in asthma. However, further investigation is warranted to address various unanswered questions and ultimately contribute to more effective clinical strategies for asthma management.

## CRediT authorship contribution statement

**Jian Han:** Conceptualization, Validation, Funding acquisition. **Hui-li Du:** Formal analysis, Writing – original draft. **Shan-shan Lu:** Visualization, Writing – original draft. **Jun-Feng Li:** Methodology, Supervision, Writing – review & editing.

## Declaration of competing interest

The authors declare no conflicts of interest.

## Appendix Supplementary materials

Image, application 1
