# Supplementary material for: Molecular mechanism of modified KAT2A-mediated histone succinylation in asthma through inhibition of ferroptosis
Source: Clinics (Sao Paulo). 2025 Sep 29;80:100786. doi: 10.1016/j.clinsp.2025.100786 (PMC12512975; doi:10.1016/j.clinsp.2025.100786)
Supplement: Supplementary file 1 [file mmc1.zip › CLINSP_CLINICS-D-25-00227.pdf]

Clinics

Molecular mechanism of modified KAT2A-mediated histone succinylation in asthma through inhibition of ferroptosis

--Manuscript Draft--

**Manuscript Number:**	CLINICS-D-25-00227R2
**Full Title:**	Molecular mechanism of modified KAT2A-mediated histone succinylation in asthma through inhibition of ferroptosis
**Article Type:**	Original Article
**Keywords:**	Asthma; Regulation; Inflammation; ferroptosis
**Corresponding Author:**	Jun-feng Li<br>Jinan Municipal Hospital Of Traditional Chinese Medicine<br>CHINA
**Corresponding Author Secondary Information:**	
**Corresponding Author's Institution:**	Jinan Municipal Hospital Of Traditional Chinese Medicine
**Corresponding Author's Secondary Institution:**	
**First Author:**	Jian Han
**First Author Secondary Information:**	
**Order of Authors:**	Jian Han
	Hui-li Du
	Shan-shan Lu
	Jun-feng Li
**Order of Authors Secondary Information:**	
**Manuscript Region of Origin:**	CHINA
**Abstract:**	Objective : KAT2A, as a kind of epigenetic enzyme has been found to be involved in ferroptosis in recent years, so this research is to explore the role and molecular role of KAT2A mediated modification of histone succinylation by inhibiting ferroptosis and its involvement in asthma.<br>Method : An asthma model was established, and the expression of KAT2A, GPX4 and SLC7A11 proteins was analyzed by Western blot and qPCR; Masson staining, TUNEL staining and HE staining were used to observe the pathological changes of lung tissues; The Clone Formation Assay and Cell Counting Kit 8 (CCK-8) were used to assess the cell viability and proliferation; and ELISA was performed for the detection of inflammatory factors; Immune cells were counted with kits. The expression of ferroptosis indicators was evaluated using qPCR and Western blot; ChIP-qPCR was performed to analyze H3K79succ and RNA pol II on the SLC7A11 promoter.<br>Results : In vitro assays verified that KAT2A regulates asthma through ferroptosis; in vivo assays verified KAT2A-mediated IL-13 succinylation modification and its effect on asthma; KAT2A controls ferroptosis via SLC7A11 and subsequently regulates asthma; Erastin significantly increased the levels of Fe2+, lipid ROS, SOD, Iron, and MDA, and decreased the expression of GPX4 and SLC7A11 and reduced inflammatory response.<br>Conclusions: We verified that KAT2A can reduce the inflammatory response caused by asthma, and further clarified that KAT2A-mediated histone succinylation modification participates in the occurrence of asthma by inhibiting ferroptosis, which may become a potential target for asthma treatment.
**Opposed Reviewers:**	
**Response to Reviewers:**	
**Additional Information:**	

Powered by Editorial Manager® and ProduXion Manager® from Aries Systems Corporation

Question	Response

Powered by Editorial Manager® and ProduXion Manager® from Aries Systems Corporation

Dear editor and reviewers

I am writing to submit the revised version of our manuscript titled “Molecular mechanism of modified KAT2A-mediated histone succinylation in asthma through inhibition of iron death”, which was previously submitted to your esteemed journal. We sincerely appreciate the time and effort that you and the reviewers dedicated to evaluating our work. Their insightful comments and constructive suggestions have been invaluable in improving the quality of our paper. A detailed point-by-point response to each reviewer’s suggestions is as follows.

Reviewer #1: General Assessment

The study investigates the role of KAT2A in asthma pathogenesis via histone succinylation-mediated regulation of ferroptosis, focusing on SLC7A11 and GPX4. The topic is innovative, linking epigenetic modifications, ferroptosis, and asthma, which holds therapeutic potential. The experimental design integrates in vitro and in vivo models, and the results suggest a promising mechanism. However, several concerns need addressing to strengthen the manuscript's validity and clarity.

Major Comments

Q1. Clarity of Hypothesis and Mechanism

- The introduction lacks a clear, concise hypothesis. While the link between KAT2A, ferroptosis, and asthma is proposed, the molecular pathway (e.g., how KAT2A-mediated succinylation directly inhibits ferroptosis via SLC7A11/GPX4) requires more detailed mechanistic explanation upfront.

Reply: Thanks to your valuable comments. We have revised the introduction section to enhance the detailed descriptions of molecular mechanisms, which we hope will help readers better understand the associations between molecules.

Q2. Experimental Design and Controls

- Sample Size and Reproducibility: The manuscript does not specify the sample size (n) for animal or cell experiments. Statistical significance claims ($p<0.01$) are

frequent but lack details on replicates or statistical tests used.

- Control Groups: Some experiments (e.g., OVA+control group) are ambiguously described. Clarify whether "OVA+control" refers to vehicle treatment or scrambled siRNA.
- Erastin's Role: The rationale for using Erastin (a ferroptosis inducer) to validate KAT2A's protective role needs elaboration. Include dose-response data or justification for Erastin concentration.

Reply: Thank you for your expert advice.

-We add a methodological section on the construction of animal models and elaborate on the details of the experiments

-The ‘OVA+control’ group represents a mouse asthma model induced with OVA and treated with vehicle at the same time, we have annotated the article.

-In this study, Erastin, as a ferroptosis inducer, was utilized to validate the protective role of KAT2A. The rationale behind this approach lies in the reverse verification of KAT2A's inhibitory effect on ferroptosis, thereby elucidating its mechanism of action in protecting against asthma. Asthma pathophysiology is associated with ferroptosis, and KAT2A is believed to be involved in the asthmatic process by inhibiting ferroptosis. As a ferroptosis inducer, Erastin promotes cellular ferroptosis. In experiments, the administration of KAT2A led to improvements in ferroptosis-related indicators, inflammatory responses, and pathological changes in cells or tissues. These improvements manifested as increased cell viability, reduced inflammatory cell infiltration, and decreased oxidative stress markers. However, the introduction of Erastin reversed these positive outcomes, resulting in decreased cell viability, increased inflammatory cells, and elevated oxidative stress indicators. This suggests that KAT2A inherently possesses the ability to inhibit ferroptosis and alleviate asthma-related symptoms. Conversely, Erastin-induced ferroptosis counteracts these protective effects of KAT2A, thereby providing reverse evidence for the hypothesis that KAT2A exerts a protective role in asthma by inhibiting ferroptosis.

3. Data Presentation and Interpretation**

- Figure Legends: Figures 1-4 are referenced but not included in the submitted content. The legends are overly brief and lack critical details (e.g., sample groups, quantification methods).

-Western Blots: Representative blots and molecular weight markers are absent. Quantification data (e.g., band intensity ratios) should be provided for GPX4, SLC7A11, and other proteins.

- Inflammatory Factors: The specific cytokines measured (e.g., IL-4, IL-13, TNF-α) are not listed, making it difficult to assess the relevance of ELISA/qPCR results.

Reply: We feel great thanks for your professional review work on our article.

-We deeply apologize for the oversight, and we have already cited the images in the corresponding positions of the manuscript.

-We have supplemented the details of the figure legend. Uncropped WB images have been uploaded as supplementary materials.

-In the introduction section, we have listed the relevant factors for detection and emphasized their relationship with asthma, enhancing the rigor of the article's structure and logic

4. Methodological Concerns

- Cell Culture Details: The origin and validation of HBE cells (e.g., ATCC certification, mycoplasma testing) are not provided.

- ChIP-qPCR: The protocol for chromatin immunoprecipitation (e.g., antibodies used, normalization controls) is insufficiently described.

- Statistical Analysis: The statistical methods (e.g., ANOVA, t-test) and software used are not specified.

Reply: Thank you for pointing out the problems in manuscript. We have revised those issues in method section.

5. Discussion Limitations

- The discussion does not adequately address how KAT2A-mediated succinylation directly modulates H3K79succ on the SLC7A11 promoter. A model diagram could help visualize the proposed pathway.
- The clinical relevance of targeting KAT2A in asthma treatment is underdeveloped. Compare findings to existing therapies (e.g., anti-IL-13 biologics) to highlight translational potential.

Reply: Thank you for your professional comment. We have modified the Discussion section based on your suggestion to add KAT2A-mediated succinylation directly modulates H3K79succ on the SLC7A11 promoter and clinical applications related to targeting KAT2A for asthma treatment.

Dear Editor,

We have revised the manuscript as requested and hope that it now meets the journal's requirements.

1.Your abstract must be structured into: Objective/Method/Results/Conclusions.

Response： We have made revisions to the abstract and structured into Objective/Method/Results/Conclusions.

2.All manuscripts must conform to specific study guidelines. Examples: Clinical Trials should follow the CONSORT Statement rules. Observational Studies (Cohort, case-control, and cross-sectional studies) should follow the STROBE Statement. Systematic Reviews and Meta-Analysis should follow the PRISMA guidelines. Diagnostic and Prognostic Studies should follow the STARD guidelines. Animal and Clinical Study should follow the ARRIVE guidelines. Please mention this in the text.

Response: Thanks for your reminder. The study involved animal experiments, we all followed the ARRIVE guidelines and have added the statement to the manuscript.

3.Please include the ORCID iD of all authors of the manuscript in the main document file.

Response: We have added the ORCID iD of all authors in revised manuscript.

4.Author contribution: CRediT statements should be provided - https://www.elsevier.com/researcher/author/policies-and-guidelines/credit-author-statement.

Response: we have added the author contribution follow the CRediT statements.

July 22, 2025

Dear Editor and Reviewers,

We are very grateful to the editors and reviewers for their valuable comments on our manuscript entitled “**Molecular mechanism of modified KAT2A-mediated histone succinylation in asthma through inhibition of ferroptosis**” and for their approval of our revisions and responses. We sincerely appreciate your feedback and constructive suggestions, which have played an indispensable role in improving the quality of our submission. Below are our responses to the reviewers' comments.

With Best Regards,

Junfeng Li

Reviewer #1

The authors have provided detailed and comprehensive responses to the reviewers' comments, addressing each point systematically. Below is an evaluation of their responses:

1.General Assessment
Clarity of Hypothesis and Mechanism

Response: The authors revised the introduction to clarify the molecular mechanisms linking KAT2A, ferroptosis, and asthma. They explicitly stated how KAT2A-mediated succinylation inhibits ferroptosis via the SLC7A11/GPX4 axis.

Evaluation: Adequate. The revised introduction now provides a clearer mechanistic pathway.

Reply：Thank you for your constructive feedback on our work and for approving our revisions and responses.

2.Experimental Design and Controls

Sample Size and Reproducibility: The authors added methodological details about sample sizes (e.g., n=3 for experiments) and statistical tests (ANOVA, Tukey’s post-test).

Control Groups: Clarified that "OVA+control" refers to vehicle treatment.

Erastin’s Role: Provided a rationale for using Erastin to validate KAT2A’s protective role via ferroptosis induction.

Evaluation: Strong improvements. The addition of experimental details enhances reproducibility.

Reply：Thank you for your constructive feedback on our work and for approving our revisions and responses.

3.Data Presentation and Interpretation

Figure Legends: Added critical details (sample groups, quantification methods) and included uncropped WB images as supplementary material.

Western Blots: Provided quantification data for GPX4, SLC7A11, etc.

Inflammatory Factors: Listed specific cytokines (IL-4, IL-13, TNF-α) and their

relevance to asthma.

Evaluation: Thorough. The revisions improve transparency and data reliability.
Reply：Thank you for your valuable feedback and positive response to our revisions and replies.

4.Methodological Concerns

Cell Culture: Confirmed HBE cell origin (Shanghai Cell Bank) and validation.

ChIP-qPCR: Detailed the protocol, including antibodies and normalization controls.

Statistical Analysis: Specified methods (ANOVA, t-test) and software (GraphPad Prism 6.0).

Evaluation: Excellent. The methods are now more rigorously described.

Reply：Thank you for your constructive feedback on our work and for approving our revisions and responses.

5.Discussion Limitations

Mechanistic Model: Added a proposed model for KAT2A-mediated H3K79succ modification on the SLC7A11 promoter.

Clinical Relevance: Compared KAT2A targeting to existing anti-IL-13 biologics, highlighting translational potential.

Evaluation: Well-addressed. The discussion now better links findings to clinical applications.

Reply：Thank you for your valuable feedback and positive response to our revisions and replies.

6.Additional Minor Revisions
1) Abstract: Restructured into Objective/Method/Results/Conclusions.

2) Guidelines Compliance: Confirmed adherence to ARRIVE guidelines for animal studies.

3) ORCIDs and CRediT: Added ORCIDs for all authors and CRediT statements for contributions.

Reply：We have revised the abstract and added relevant information to meet the journal's publication requirements.

Overall Assessment

The authors have diligently addressed all reviewer concerns, enhancing the manuscript’s clarity, rigor, and translational relevance. The revisions align with journal standards, and the responses demonstrate a commitment to improving the study’s quality. The manuscript is now significantly strengthened and ready for publication after minor review.

Thank you again for your evaluation of our manuscript. We look forward to the article entering the acceptance process. Please feel free to contact me if you have any questions.

Figure

Click here to access/download;Figure;Figure.pptx

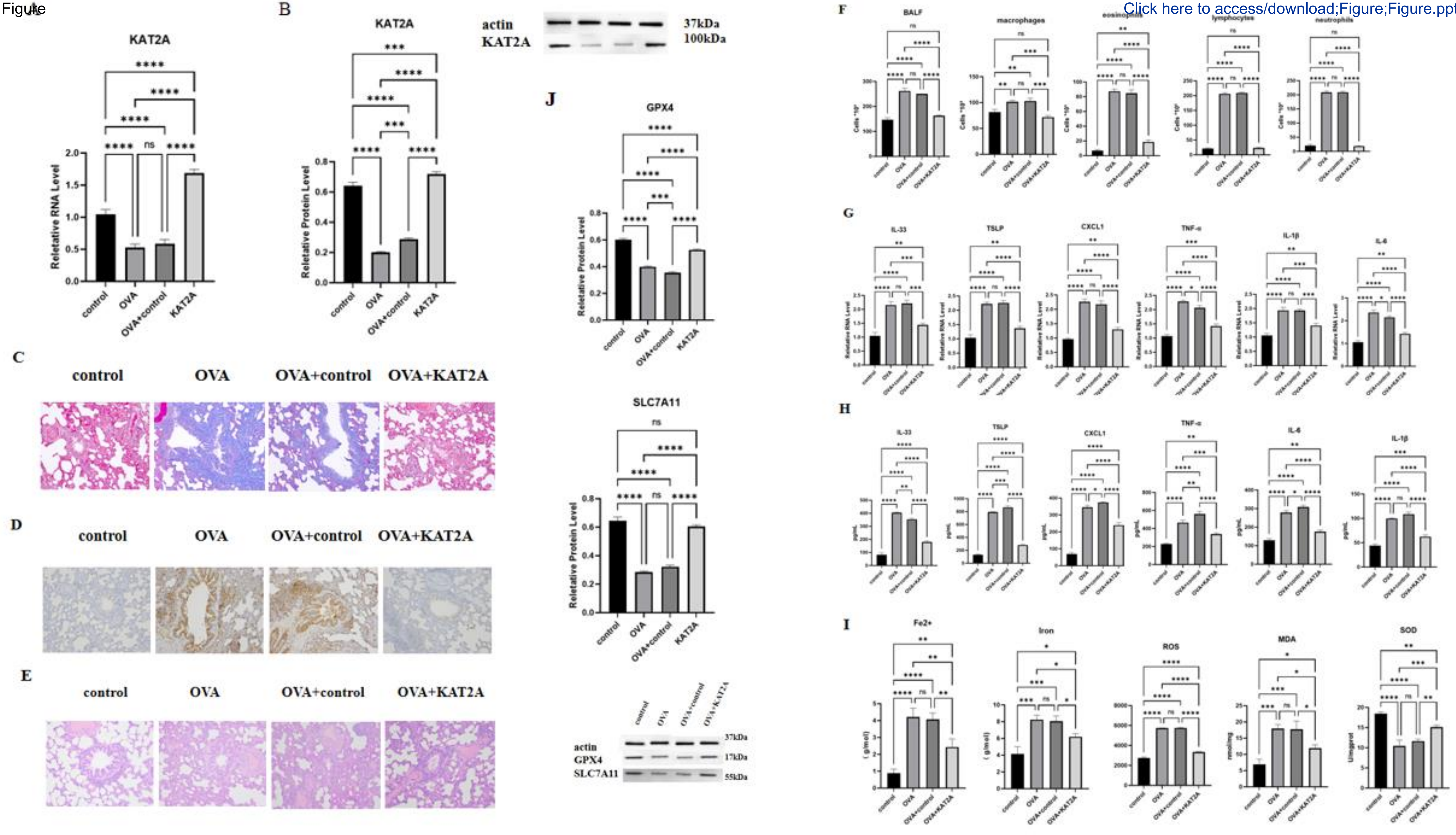

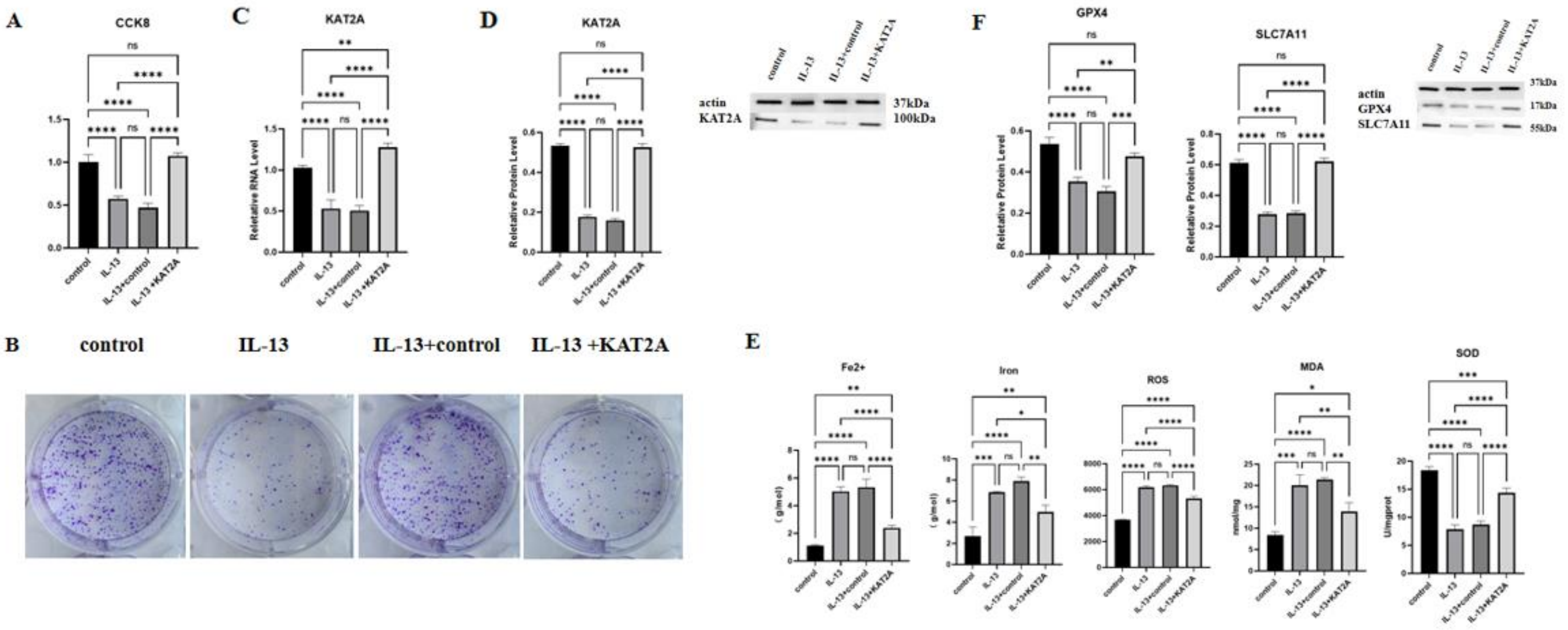
A
CCK8
C
KAT2A
Reletative RNA Level
D
KAT2A
Reletative Protein Level
control
IL-13
IL-13+control
IL-13+KAT2A
actin
KAT2A
37kDa
100kDa
F
GPX4
SLC7A11
Reletative Protein Level
actin
GPX4
SLC7A11
37kDa
17kDa
55kDa
B
control
IL-13
IL-13+control
IL-13 +KAT2A
E
Fe2+
Iron
ROS
MDA
SOD
(g/mol)
nmol/mg
U/mgprot
ns
****
***
**
*

Figure

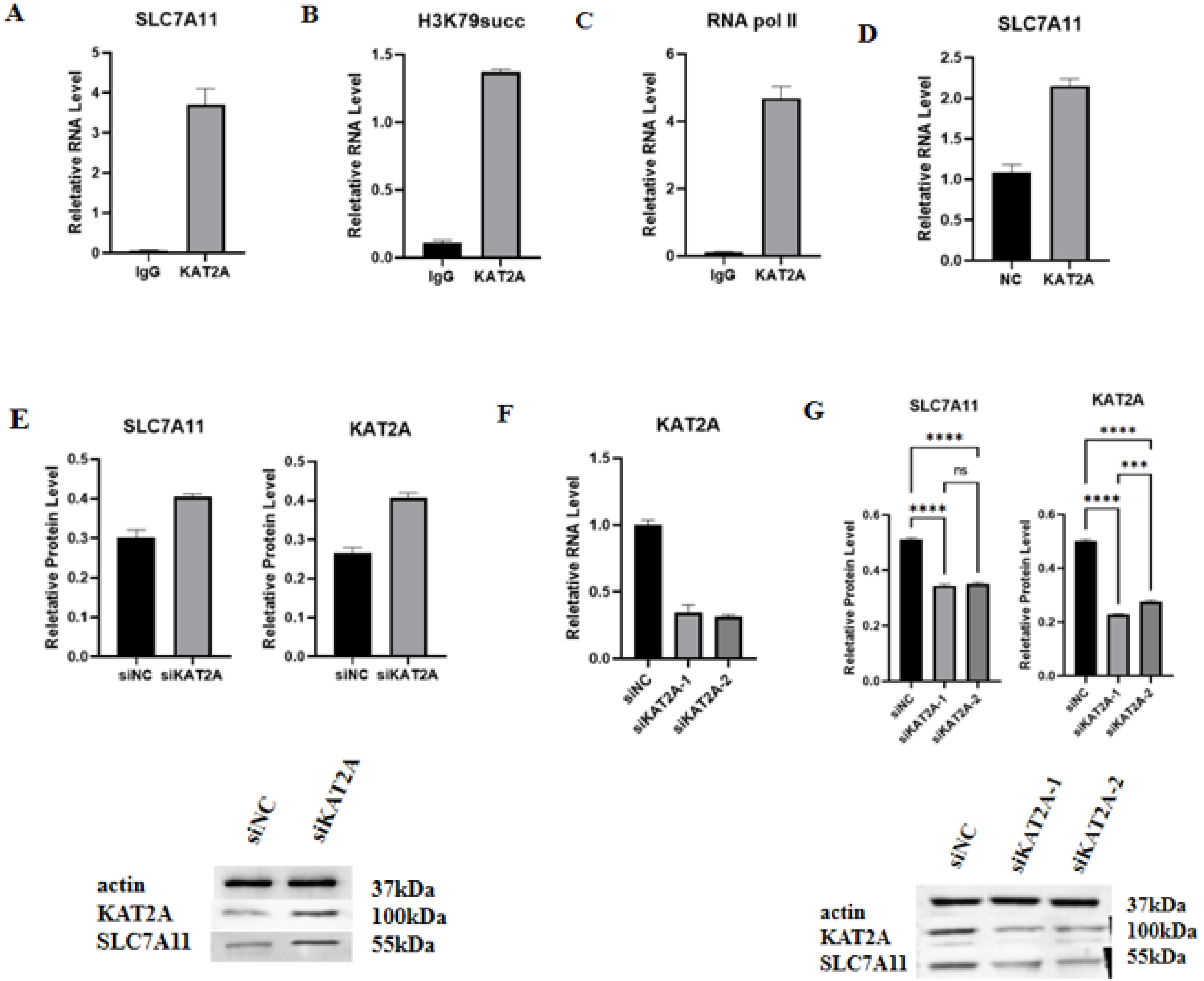
A
SLC7A11
Reletative RNA Level
IgG
KAT2A
B
H3K79succ
Reletative RNA Level
IgG
KAT2A
C
RNA pol II
Reletative RNA Level
IgG
KAT2A
D
SLC7A11
Reletative RNA Level
NC
KAT2A
E
SLC7A11
Reletative Protein Level
siNC
siKAT2A
KAT2A
Reletative Protein Level
siNC
siKAT2A
F
KAT2A
Reletative RNA Level
siNC
siKAT2A-1
siKAT2A-2
G
SLC7A11
Reletative Protein Level
ns
KAT2A
Reletative Protein Level
siNC
siKAT2A
actin
KAT2A
SLC7A11
37kDa
100kDa
55kDa
siNC
siKAT2A-1
siKAT2A-2
actin
KAT2A
SLC7A11
37kDa
100kDa
55kDa

Figure

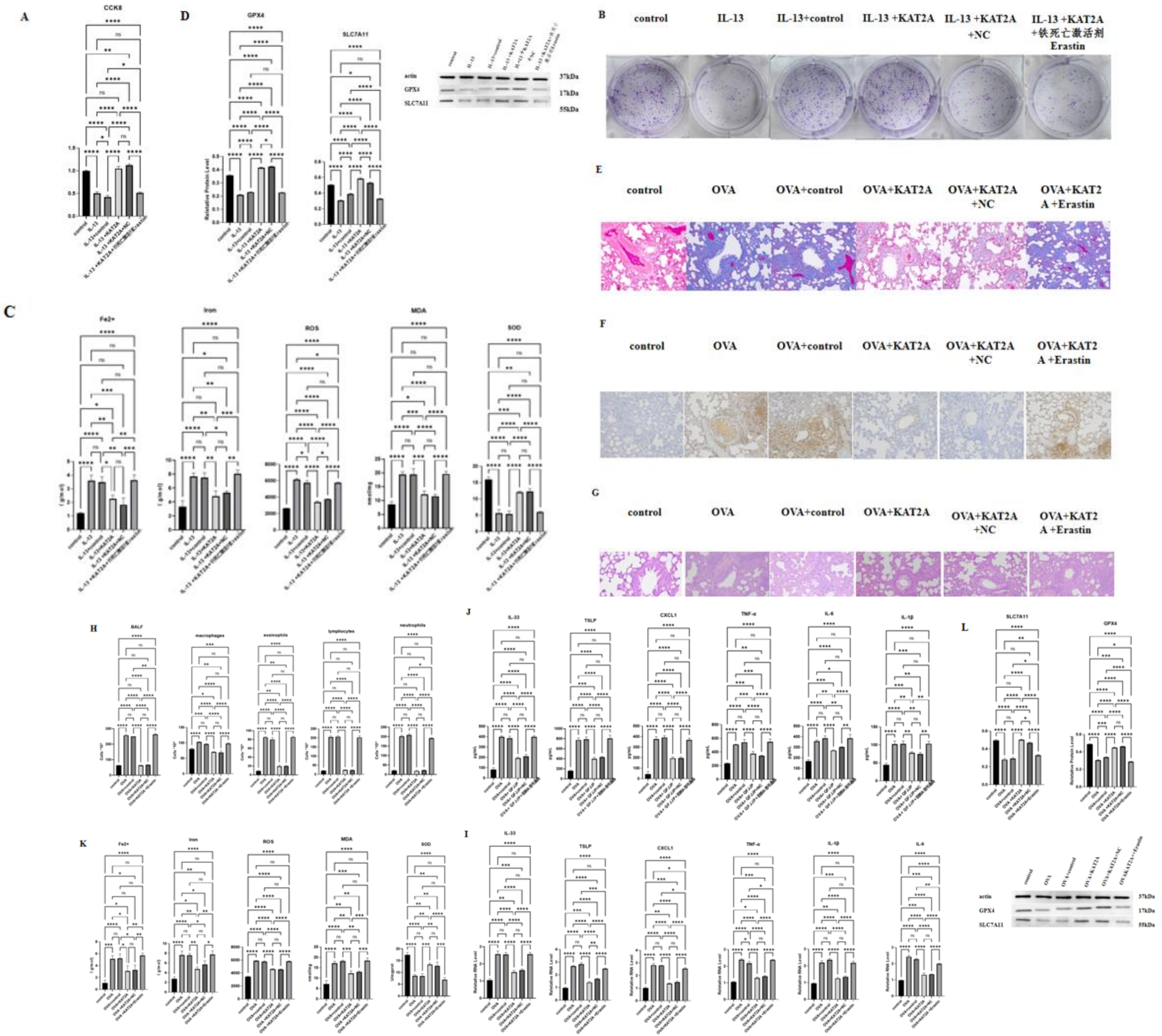

A
CCK8
D
GPX4
SLC7A11
actin
GPX4
SLC7A11
37kDa
17kDa
55kDa
Relative Protein Level
B
control
IL-13
IL-13+control
IL-13 +KAT2A
IL-13 +KAT2A +NC
IL-13 +KAT2A +铁死亡激活剂 Erastin
C
Fe2+
Iron
ROS
MDA
SOD
E
control
OVA
OVA+control
OVA+KAT2A
OVA+KAT2A +NC
OVA+KAT2 A +Erastin
F
control
OVA
OVA+control
OVA+KAT2A
OVA+KAT2A +NC
OVA+KAT2 A +Erastin
G
control
OVA
OVA+control
OVA+KAT2A
OVA+KAT2A +NC
OVA+KAT2 A +Erastin
H
BALF
macrophages
eosinophils
lymphocytes
neutrophils
J
IL-33
TSLP
CXCL1
TNF-α
IL-6
IL-1β
L
SLC7A11
GPX4
K
Fe2+
Iron
ROS
MDA
SOD
I
IL-33
TSLP
CXCL1
TNF-α
IL-1β
IL-6
actin
GPX4
SLC7A11
37kDa
17kDa
55kDa

Figure

Figure

Click here to access/download;Figure;figure-4-1.tif

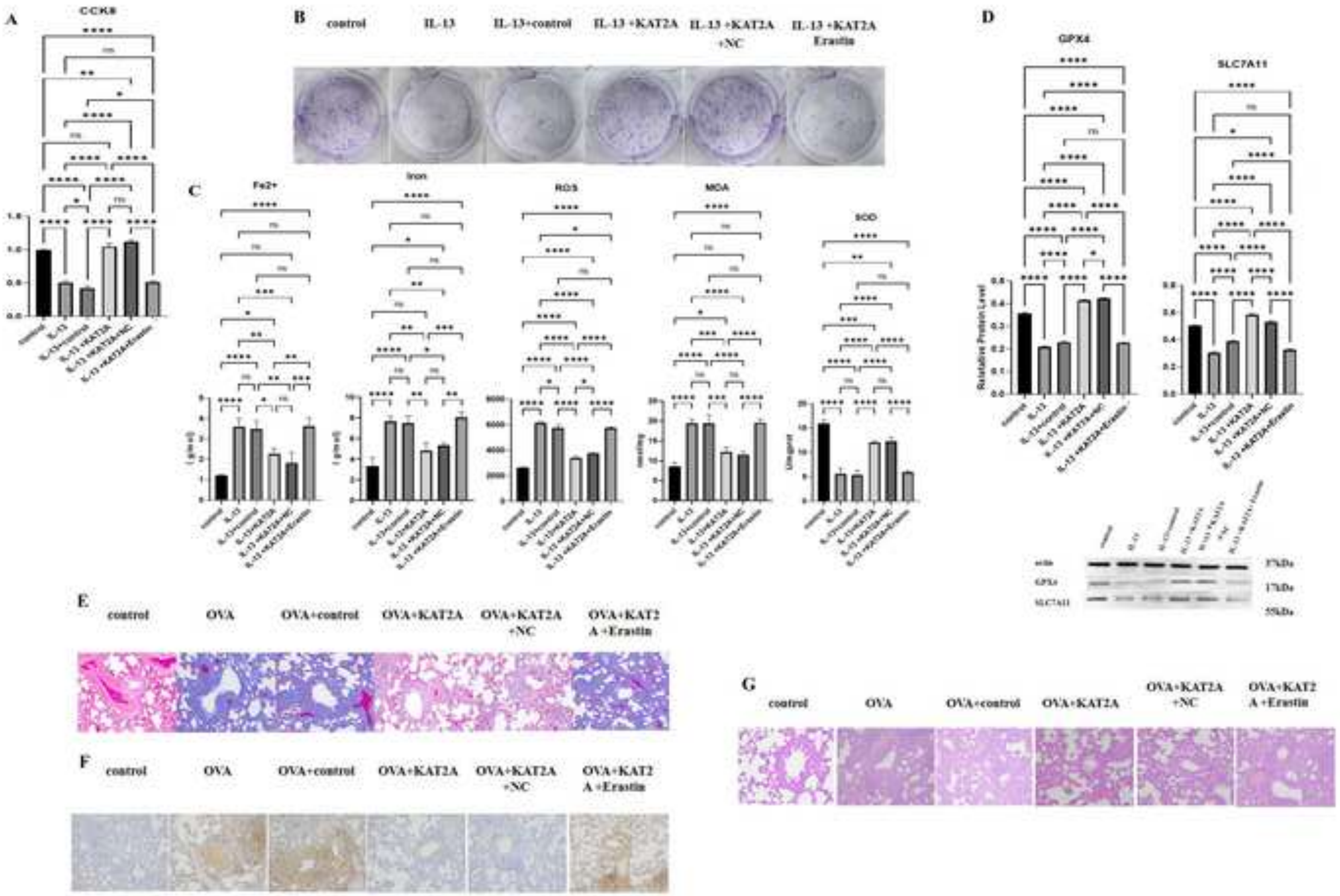

Figure Click here to access/download;Figure;figure-1.tif

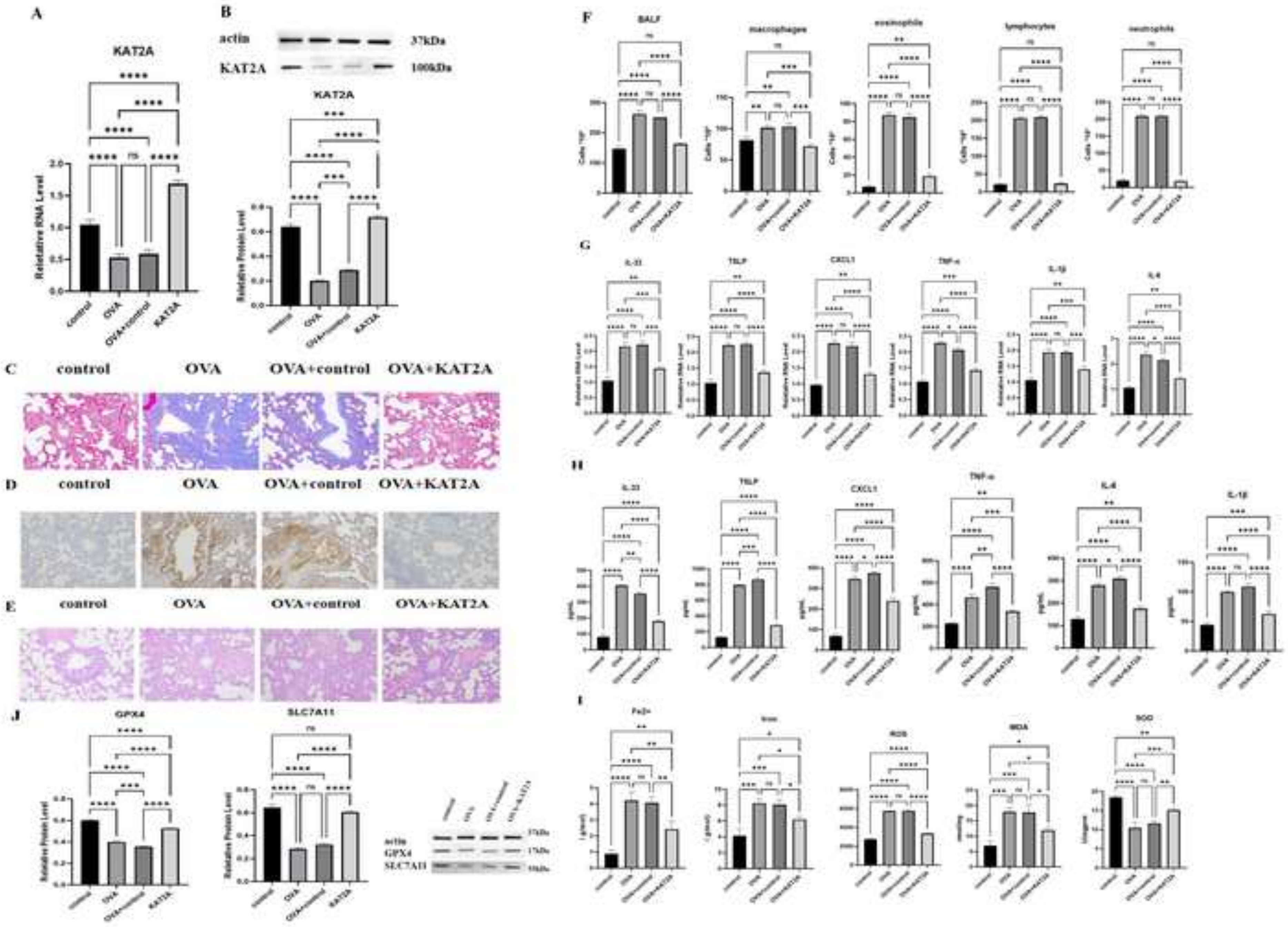

Figure

Click here to access/download;Figure;figure-2.tif

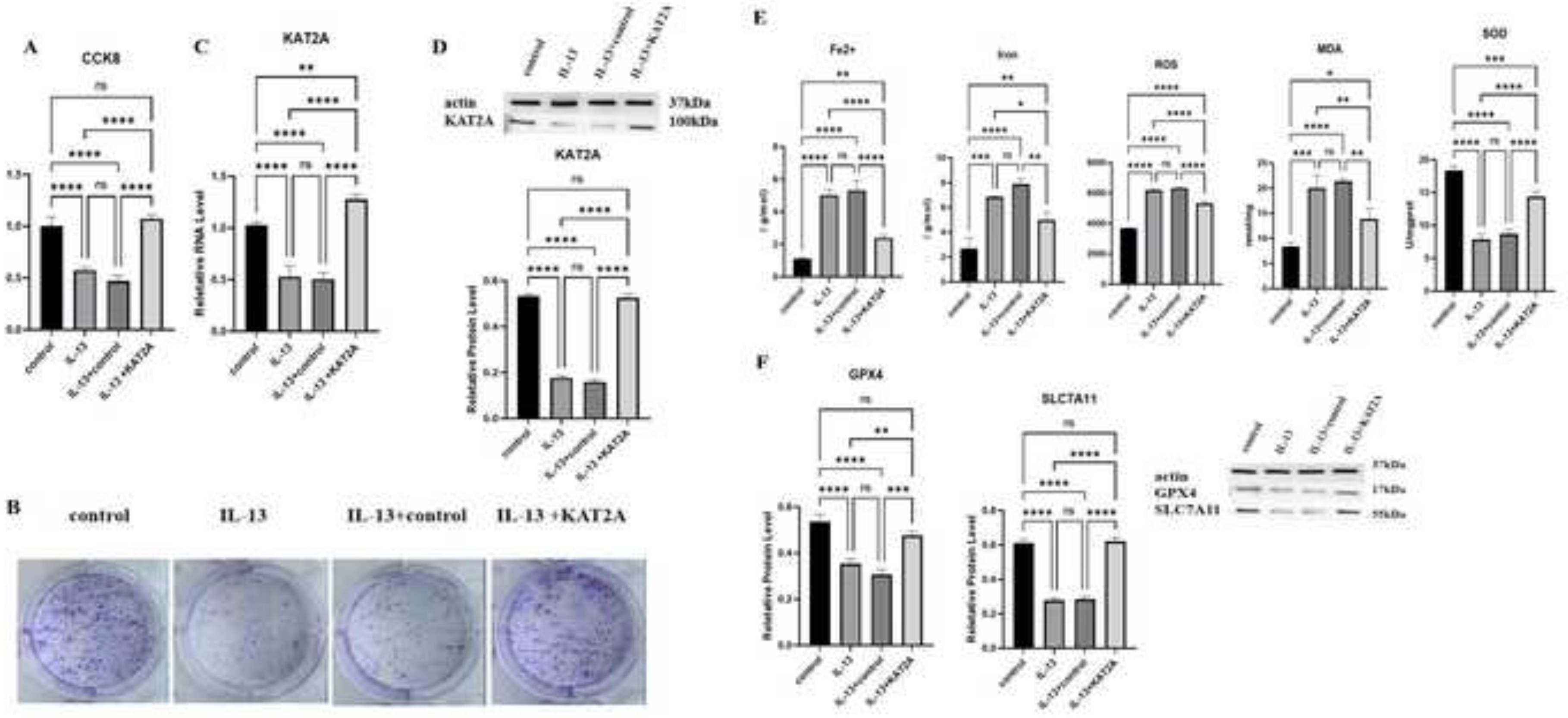

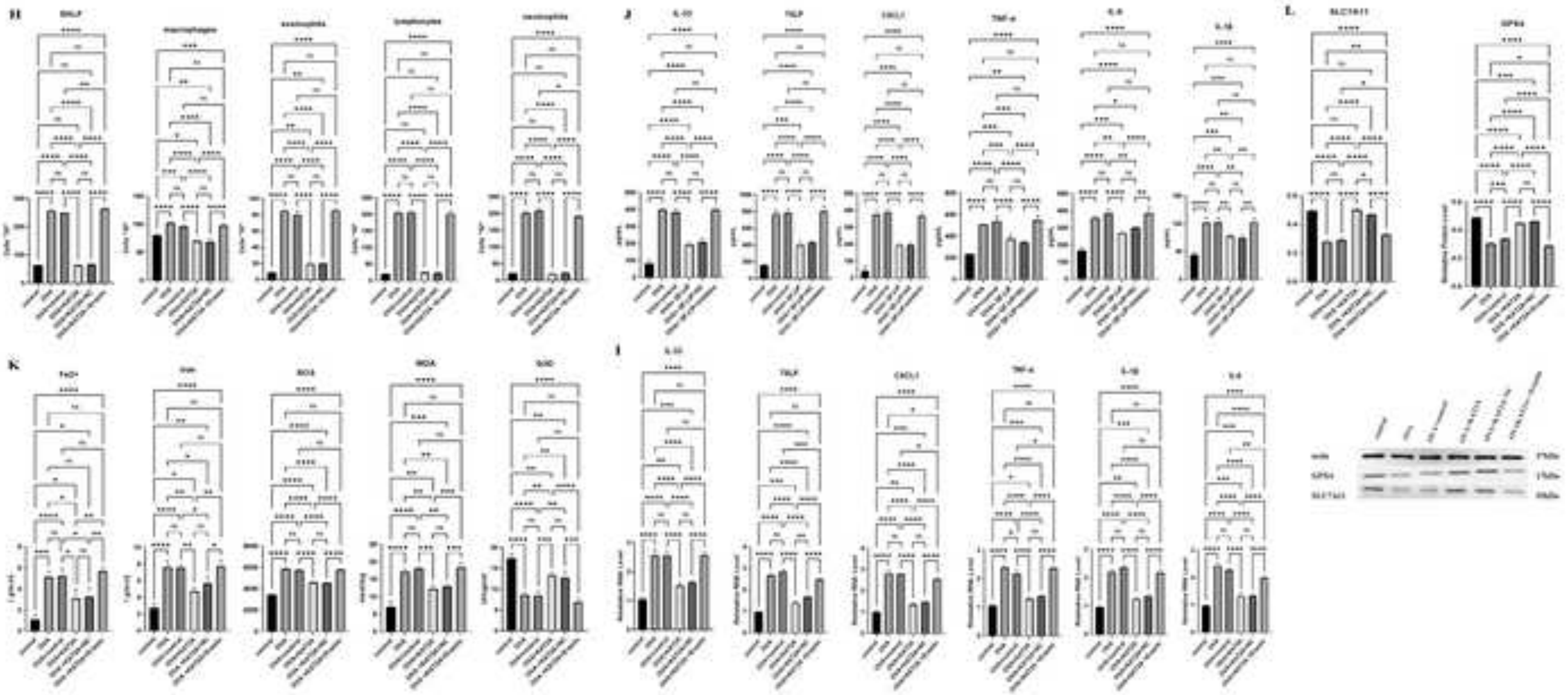

Figure

Click here to access/download;Figure;figure-3.tif

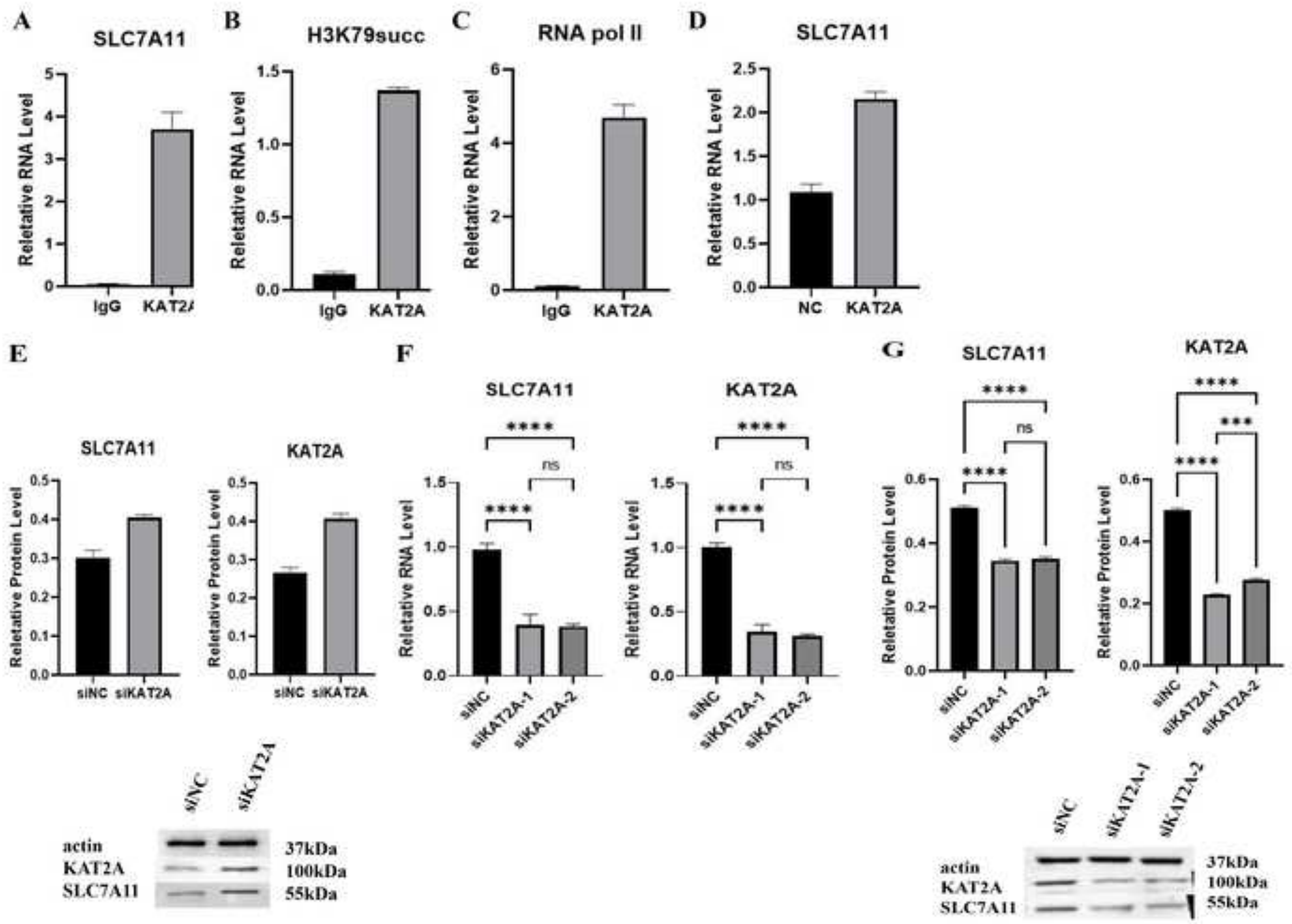

Highlights

- KAT2A, epigenetic enzyme has been found to be involved in iron death
- Asthma model shown the expression of KAT2A, GPX4 and SLC7A11
- Erastin significantly increased the levels of $Fe2+$, lipid ROS, SOD, Iron, and MDA
- Decreased the expression of GPX4 and SLC7A11 and reduced inflammatory response
- KAT2A-mediated histone succinylation by inhibiting iron death, provide therapeutic

Click here to access/download
Supplementary Material
Supplementary materials-WB uncut figures.rar

Molecular mechanism of modified KAT2A-mediated histone succinylation in asthma through inhibition of ~~iron death~~ferroptosis

Jian Han1, Hui-li Du2, Shan-shan Lu3, Jun-feng Li 4,*

1.Department of Respiratory and Critical Care Medicine, Affiliated Hospital of Shandong University of Traditional Chinese Medicine, Shandong Province Hospital of Traditional Chinese Medicine, Jinan, 250011, China

2.Department of Respiratory and Critical Care Medicine, Feicheng People's Hospital, Taian, 271600, China

3.Disinfection supply center, Guang'anmen Hospital Jinan Hospital (Jinan Municipal Hospital of Traditional Chinese Medicine), Jinan, 250012, China

4.Pediatrics of traditional Chinese medicine, Qingdao Women and Children's Hospital, Qingdao, 266000, China.

1: email: 60011703@sdutcm.edu.cn , ORCID: 0009-0002-89-391-0626
2: email: huilidu1986@163.com , ORCID: 0009-0005-2767-8334
3: email: 316886281@qq.com , ORCID: 0009-0008-0104-5135
*Correspondence: Jun-feng Li: email: lijunfeng0008@163.com , ORCID: 0009-0006-7057-9777

Abstract

Objective: KAT2A, as a kind of epigenetic enzyme has been found to be involved in ferroptosis in recent years, so this research is to explore the role and molecular role of KAT2A mediated modification of histone succinylation by inhibiting ferroptosis and its involvement in asthma.

Method: An asthma model was established, and the expression of KAT2A, GPX4 and SLC7A11 proteins was analyzed by Western blot and qPCR; Masson staining, TUNEL staining and HE staining were used to observe the pathological changes of lung tissues; The Clone Formation Assay and Cell Counting Kit 8 (CCK-8) were used to assess the cell viability and proliferation; and ELISA was performed for the detection of inflammatory factors; Immune cells were counted with kits. The expression of ferroptosis indicators was evaluated using qPCR and Western blot; ChIP-qPCR was performed to analyze H3K79succ and RNA pol II on the SLC7A11 promoter.

Results: In vitro assays verified that KAT2A regulates asthma through ferroptosis; in vivo assays verified KAT2A-mediated IL-13 succinylation modification and its effect on asthma; KAT2A controls ferroptosis via SLC7A11 and subsequently regulates asthma; Erastin significantly increased the levels of Fe2+, lipid ROS, SOD, Iron, and MDA, and decreased the expression of GPX4 and SLC7A11 and reduced inflammatory response.

Conclusions: We verified that KAT2A can reduce the inflammatory response caused by asthma, and further clarified that KAT2A-mediated histone succinylation modification participates in the occurrence of asthma by inhibiting ferroptosis, which may become a potential target for asthma treatment.

Keywords: Asthma; Regulation; Inflammation; ~~Iron death~~ferroptosis

1. **Introduction**

Asthma, a common chronic inflammatory airway disease characterized by airway inflammation, remodeling, and hyperresponsiveness, significantly impacts the quality of life and health status of a large global population[1]. According to the World Health Organization, approximately 235 million people worldwide suffer from asthma, with varying prevalence rates across different regions. In some high-incidence areas such as Australia, the prevalence rate can reach up to 21%[2]. In China, the number of asthma patients has exceeded 45 million, with a prevalence rate of approximately 4.2%[3]. Due to factors like environmental pollution, smoking, allergies, and population aging, the incidence and mortality rates of asthma are gradually increasing, posing a heavy medical burden on individuals and society[4]. Therefore, it is urgent to deeply explore the pathophysiology of asthma and identify effective therapeutic targets.

The pathophysiology of asthma is highly complex, involving multiple cellular and molecular mechanisms. Recently, the role of ferroptosis, a novel cell death mode, in the pathogenesis of asthma has garnered increasing attention[5, 6]. Ferroptosis is a form of cell death triggered by lipid peroxidation, where iron accumulation leads to increased generation of lipid reactive oxygen species (ROS), ultimately damaging the cell membrane structure[7]. In the airway epithelial cells of asthma patients, key components of the ferroptosis defense system, such as cystine/glutamate antiporter SLC7A11 and glutathione peroxidase 4 (GPX4), play crucial roles. SLC7A11 mediates cystine uptake to maintain GPX4 activity, thereby neutralizing lipid peroxides and inhibiting

Formatted: Indent: First line: 0 ch

Formatted: Font: (Default) Times New Roman

Formatted: Font: (Default) Times New Roman

Formatted: Font: (Default) Times New Roman

Formatted: Font: (Default) Times New Roman

Formatted: Font: (Default) Times New Roman

Formatted: Font: (Default) Times New Roman

ferroptosis, Hhowever, when SLC7A11 and GPX4 functions are impaired, ferroptosis can propagate in airway epithelial cells. Studies have found reduced SLC7A11/GPX4 expression and elevated levels of the lipid peroxidation marker 4-HNE in bronchial biopsy samples from patients with severe asthma, indicating a close association between decreased anti-ferroptosis capacity and asthma severity.

[8, 9]. However, when SLC7A11 and GPX4 functions are impaired, ferroptosis can propagate in airway epithelial cells. Studies have found reduced SLC7A11/GPX4 expression and elevated levels of the lipid peroxidation marker 4-HNE in bronchial biopsy samples from patients with severe asthma, indicating a close association between decreased anti-ferroptosis capacity and asthma severity.

Lysine acetyltransferase 2A (KAT2A), also known as general control non-repressible protein 5 (GCN5), is an important epigenetic enzyme belonging to the GNAT family. It alters DNA structure by transferring acetyl groups, enhancing the transcription level of specific DNA, and regulating various biological processes[10]. Recent studies have revealed that KAT2A plays a key role in regulating cellular redox homeostasis. Besides its classical function in histone acetylation, KAT2A can directly modify metabolic enzymes in the glutathione synthesis pathway through succinylation[11]. Mounting evidence suggests that KAT2A may participate in the development of various diseases by regulating ferroptosis, although its specific mechanistic role in asthma remains elusive.

Currently, it is hypothesized that KAT2A-mediated succinylation may directly inhibit ferroptosis via the SLC7A11/GPX4 axis. As an enzyme capable of regulating gene transcription, KAT2A may modify the promoter region of the SLC7A11 gene, influencing its transcription process. Specifically, KAT2A could catalyze histone succinylation, altering chromatin structure and facilitating the binding of transcription factors to the SLC7A11 gene promoter region, thereby promoting SLC7A11 expression. Elevated SLC7A11 expression enables increased cystine uptake, maintaining intracellular glutathione (GSH) levels and providing sufficient substrates for GPX4, enhancing its activity and effectively inhibiting lipid peroxidation and ferroptosis. However, this hypothesis requires further experimental validation.

To clarify the role and mechanism of KAT2A in asthma, a series of experiments were conducted in this study. Regarding the detection of inflammatory factors, given the involvement of multiple cytokines in the inflammatory response during asthma pathogenesis, and based on numerous previous studies demonstrating the key roles of interleukin-4 (IL-4), interleukin-13 (IL-13), and tumor necrosis factor-alpha (TNF-α) in asthma inflammation, these cytokines were selected for investigation[12, 13]. IL-4 promotes IgE production by B cells, playing a crucial role in allergic reactions and airway inflammation in asthma; IL-13 induces high secretion of airway mucus, promotes airway smooth muscle contraction and remodeling; TNF-α activates inflammatory cells, facilitating the release of inflammatory mediators and exacerbating airway inflammation.

this experimentWe validate the protective effect of KAT2A through Erastin. As an inducer of ferroptosis,

The purpose of using Erastin in this experiment was to validate the protective effect of KAT2A. iIf KAT2A indeed exerts a protective effect on asthma by inhibiting ferroptosis, the introduction of Erastin to induce ferroptosis should weaken or reverse this protective effect[14]. Specifically, in vitro experiments are expected to show a decrease in cell viability and proliferative

Formatted: Font: (Default) Times New Roman

Formatted: Font: (Default) Times New Roman

Formatted: Font: (Default) Times New Roman

Formatted: Font: (Default) Times New Roman

Formatted: Font: (Default) Times New Roman

Formatted: Font: (Default) Times New Roman

capacity in the KAT2A overexpression group upon Erastin exposure, despite initial enhancement by KAT2A. Ferroptosis-related indicators (such as Fe^{2+}, lipid ROS, and MDA levels) are anticipated to increase, while GPX4 and SLC7A11 expression should decrease, reversing the initial beneficial effects of KAT2A. Additionally, the expression of inflammatory cytokines like IL-4, IL-13, and TNF-α, initially reduced by KAT2A, is expected to increase upon Erastin treatment. In vivo experiments are expected to demonstrate that the pathological changes in lung tissue, such as inflammatory cell infiltration and airway remodeling, improved by KAT2A intervention, will be exacerbated again upon Erastin administration. Similarly, BALF levels of inflammatory cytokines (IL-4, IL-13, and TNF-α) and inflammatory cell counts, initially reduced by KAT2A, are anticipated to increase with Erastin treatment. Through these experimental results, we aim to further elucidate the specific mechanism by which KAT2A-mediated histone succinylation modifies and inhibits ferroptosis, thereby participating in the asthmatic process. This will provide new potential targets and theoretical basis for asthma treatment. Asthma is a common chronic inflammatory disease of the respiratory system characterized by airway inflammation, airway remodeling and airway hyperresponsiveness [1]. According to the World Health Organization, 235 million people worldwide suffer from asthma, with prevalence rates not being the same in different regions; the global prevalence rate is about 4.3%, and the prevalence rate in areas with high prevalence of asthma (e.g., Australia) even reaches 21% [2]. Recent studies have shown that more than 45 million people in China suffer from asthma, with a prevalence rate of about 4.2%, and 15.5% of asthma patients require emergency treatment and 7.2% require hospitalization every year [3]. With the process of air pollution, smoking, allergies, and aging of the population, the morbidity and mortality of asthma are gradually increasing[4]. Therefore, asthma imposes a heavy burden on individuals and national health services, and how to effectively promote asthma prevention and treatment has become a public health and healthcare issue that we need to seriously face and solve

Asthma belongs to the lung diseases, and its clinical manifestations include wheezing, cough, sputum, chest tightness, and dyspnea [5]. Its pathogenesis and mechanisms are very complex, and many factors are involved, such as: host factors, environmental factors, exercise factors, climatic factors, dietary factors, drug factors, socioeconomic status, literacy, and psycho-emotional factors, etc[6][7], of which the most important risk factors are host factors (susceptibility factors, such as genetic predisposition, allergy, and obesity, etc.) and environmental factors (important factors, such as allergens, air pollution, dust, smoking, etc.) [8]. Currently asthma is not curable and the most important therapeutic goal is to achieve good control of the symptoms and thus reduce the number of attacks, with the main principles of treatment being anti-inflammatory and bronchodilator [9]. Asthma is strongly associated with inflammatory cell infiltration and AHR [10]. Meanwhile, the accumulation of large amounts of lipid peroxides is a marker of iron death, suggesting that iron death may be associated with asthma. A recent study showed that high iron levels in lung cells are associated with worsening asthma, in which high levels of iron lead to airway inflammation and an over-oxidized state, resulting in iron death [11].

Formatted: Font: Not Superscript/ Subscript

Lysine Acetyltransferase 2A (KAT2A), also known as general control non-repressor protein 5 (GCN5), was the first acetyltransferase to be characterized and is a key enzyme belonging to the GNAT family of epigenetic enzymes [12]. It alters the structure of DNA through the transfer of acetyl groups, which enhances the transcriptional level of specific DNA and selectively produces various traits [13].KAT2A can enhance the transcriptional activity of downstream genes by

regulating histone acetylation, histone succinylation, and recruiting transcriptional co-stimulatory factors [14][15].KAT2A has a strong de-succinimidatory enzyme activity in the mitochondria. Unlike other family members, the deacetylase activity of KAT2A is extremely low [16]. In recent years, studies have also identified KAT2A as a key enzyme in lysine modification (mainly able to participate in succinylation, malonylation, and glutaralylation) [17]. It has also been shown that KAT2A knockdown significantly upregulates the level of succinylation modification of key enzymes in processes such as cellular metabolism and oxidative stress [18]. In addition to its basic transcriptional regulatory functions, the acetylation of KAT2A is closely related to the maintenance of genome stability, cell differentiation of stem cells, cell proliferation, inflammation, aging, oxidative stress, energy metabolism, adipose transformations, and disease genesis [19]. Zhang Y [20] et al. study demonstrated that the acetyltransferase KAT2A permits metabolic and epigenetic reprogramming of NLRP3 inflammasome activation in inflammatory macrophages, and thus targeting KAT2A represents a potential therapeutic approach for patients suffering from RA and related inflammatory diseases.

In this study, we explored the role of KAT2A-mediated histone succinylation modification involved in asthma through inhibition of iron death and determined the mechanism of its function.

2. Materials and methods

2.1 Cell culture and Cell transfection and treatment

HBE cells were purchased from the Shanghai Cell Bank of the Chinese Academy of Sciences (Shanghai, China) and maintained in a 37°C incubator containing fetal bovine serum (10% of FBS; BI, China) and 1% streptomycin/penicillin (Sigma, USA) filled with 5% CO_2. The siRNA targeting KAT2A gene was ordered from RiboBio (Guangzhou, China). siRNA was transfected into cells with PEI reagent and Lipofectamine RNAiMAX and kept for 48 hrs. After that, cells were treated with cycloheximide (CHX, 200 ng/mL) for 24 hours to inhibit protein synthesis. pCDNA3.1 for the construction of the KT2A overexpression vector was purchased from Thermo Fisher Scientific Inc. (USA).

2.2 Construction of an OVA-induced asthma mouse model

A total of 32 male C57BL/6J mice (6-8 weeks) were procured from Beijing Vital River Laboratory Animal Technology Co., Ltd. Except for 10 controls, the other mice were challenged by intraperitoneal injection of 0.2 ml of saline solution containing 20μg of OVA and 2 mg of aluminum hydroxide on day0 and day7，From days14 to16, mice were inhaled with 3% OVA solution by ultrasonic nebulizer. for 30 minutes each time. Mice were challenged with 3% OVA on days 21 to 23. All animal studies follow the ARRIVE guidelines and the ethics batch number is MDL2023-10-19-01.

2.3 Quantitative real time PCR

Total RNA was extracted from cells using Trizol reagent (Thermo, USA) and reverse transcribed to cDNA by using First-strand synthesis kit (Transgene, China). RNA level was measured using a SYBR Green Supermix (Transgene) and detected using the Real-Time PCR Detection System (Bio-Rad, USA). The β-Actin level was set as internal reference for normalization.

2.4 Western blot assay

Total proteins from the mouse mullerHBE cells were exctrtaed by using lysis bffer and then were separated using SDS-PAGE. After that the protein in the gel were transferred into membranes.

Formatted: Font: Not Superscript/ Subscript

Formatted: Font: Not Superscript/ Subscript

Formatted: Font: Not Superscript/ Subscript

Formatted: Subscript

Formatted: Font: (Default) Times New Roman, 10.5 pt, Font color: Auto, Pattern: Clear

Formatted: Font: Bold

Formatted: Font: 10.5 pt

Formatted: Font: 10.5 pt

Formatted: Font: (Default) Times New Roman, Not Highlight

Formatted: Font: (Default) Times New Roman, Not Highlight

Formatted: Font: 10.5 pt

Formatted: Font: 10.5 pt

Formatted: Font: (Default) Times New Roman, Not Highlight

Formatted: Font: (Default) Times New Roman

After blocking in 5% non-fat milk, protein bands were probed with antibodies for caspase3, LC3, p62, pAKT, AKT,p-mTOR,mTOR, and β-actin overnight at 4°C℃. After that, the membrane was immersed in secondary antibody conjugated HRP and then wash the membrane with washing buffer, Finally, ECL reagent ws added to see the protein bands. All antibodies were procured from Abcam (USA).

2.5 Chromatin immunoprecipitation (ChIP) assay

The ChIP Assay Kit (P2078, Beyotime) was applied for ChIP detection. Chromatin was cross-linked in 1% formaldehyde at 37℃ for 10 min, sonicated to a size of 200-1000 bp, and then incubated with anti-SLC7A11/H3K79succ/RNA pol II for 8 h at 4℃, followed by incubated with Protein A+G Agarose for 1 h at 4℃. After elution of the protein/DNA complex. The DNA was de-crosslinked. Immunoprecipitated DNA was analyzed by RT-qPCR.

RNA polymerase II：abcam，ab300575

SLC7A11：abcam，ab302919

H3K79succ：PTM BIO，PTM-412

2.6 CCK-8 assay

The cells in sh-NC and sh-METTL3 groups were treated with different concentration gradient of Cisplatin, and CCK-8 assay was performed 24 h later. The cells were incubated at 37 °C for 4 h in a 5% CO2 incubator, the supernatant was discarded, and the cells were incubated with DMSO for 10 min, then the absorbance at 450 nm was measured on an enzyme counter, and the cell activity was calculated.

2.7 Histochemical staining

Immunohistochemical staining: xylene I dewaxing for 10 minutes. Xylene II dewaxing for 10 minutes. Gradient alcohol hydration: 100% alcohol, 95% alcohol, 80% alcohol, 70% alcohol. Soak in each gradient of alcohol for 5 minutes. The sections were rinsed in running water for 10 min. and in PBS 3 times for 5 min each. Place sections in prepared sodium citrate solution and thaw in microwave oven for 20 minutes. Cool at room temperature. Rinse 3 times with PBS for 5 minutes each time. Add 0.3% hydrogen peroxide solution dropwise on top of the tissue and incubate at 4°C for 15 minutes. The tissue was rinsed 3 times with phosphate buffer solution at 5-minute intervals. Incubate with 10% goat serum for 30 min at 37°C. Dilute USP37 antibody with PBS, add appropriate amount of USP37 antibody dilution to each tissue, and incubate at 4°C overnight. run 3 times in PBS, each time 5 minutes apart. Appropriate amount of biotin secondary antibody was added to each tissue dropwise and incubated at 4°C for 30 min. and washed with PBS for 3 times, each time with an interval of 5 min. Horseradish peroxidase was added dropwise to the tissue and incubated at 4°C for 30 min. and washed with PBS for 3 times, each time with an interval of 5 min. The color was developed by adding DAB colorant and rinsed in PBS for 10 minutes. Sections were placed in hematoxylin for 2 minutes, soaked in warm water and rinsed in PBS for 10 minutes. Gradient alcohol 70% alcohol, 80% alcohol, 95% alcohol, 100% alcohol. Each soaked for 5 minutes.

2.8 Masson staining

Masson staining also followed the kit guidelines, and was performed sequentially with ferric hematoxylin staining for 5 min, water washing, 1% hydrochloric acid ethanol staining for 1 s, water washing, Li Chun red staining for 10 min, phosphomolybdic acid staining for 1~5 min, and toluidine blue water washing, and finally washed with 1% glacial acetic acid for 1 min and dehydrated and sealed, and was observed under a microscope and photographed with pathological

Formatted: Font: Bold

Formatted: Font: Not Italic

Formatted: Font: Bold

Formatted: Font: (Default) Times New Roman, Not Highlight

Formatted: Font: (Default) Times New Roman, Not Highlight

Formatted: Font: (Default) Times New Roman, Font color: Auto, Not Highlight

Formatted: Font: (Default) Times New Roman, Not Highlight

Formatted: Font: (Default) Times New Roman, Not Highlight

Formatted: Font: (Default) Times New Roman, Not Highlight

Formatted: Font: (Default) Times New Roman, Not Highlight

Formatted: No bullets or numbering

pictures (with a magnification of 200×), and the percentage of the stained area to the total area was calculated with Image J fiber to calculate the percentage of stained area to total area.

2.9 TUNEL staining

Cells were seeded on the bottom of transwell 6-well plates with cell crawlers to construct a co-culture model. After intervention, TUNEL staining was performed: discard the original culture medium, add fixative and incubate at room temperature for 20 min, add membrane-breaking solution and incubate for 5 min, add buffer and incubate for 10 min, take an appropriate amount of TDT enzyme and incubate at 37°C for 1 h. Add DAPI staining solution and incubate for 10 min, avoiding light, and then seal the plate. Observe and collect images under fluorescence microscope.

2.10 MDA assay for lipid peroxidation levels

Cells were collected and operated according to the kit instructions. Each well absorbance was measured using a multifunctional enzyme marker and MDA level was calculated.

2.10.1 Fe^{2+} content assay

Cells were collected and operated according to the kit instructions. Determine the absorbance value of each well using a multifunctional enzyme marker and calculate the Fe2+ content according to the standard curve.

2.10.2 ROS level

The procedure was performed according to the instructions of the Reactive Oxygen Kit. Cells were collected and resuspended, DCFH-DA was added, mixed and incubated at 37ºC for 20 min. Fluorescence intensity was detected by flow cytometry and analyzed by FlowJo software.

2.11 ELISA

Blank, standard, and sample group was tested with ELISA assay. Briefly, 50 μL of standard samples was added on the enzyme-labeled coating plate. For samples to be tested, 40 μL of sample dilution was added to the sample group, and then 10 μL of samples to be tested were added, and then gently shaken well. Incubate at 37 °C for 30 min and then wash with detergent, and repeat 5 times to remove the tissue solution. Add developer A and developer B to each group, shake gently, and kept at 37 °C for 10 min in the dark, return the blank group to 0, and measure the absorbance at 450 nm (A) of each group in turn. Three samples were measured in each group, and each sample was tested three times.

2.12 Clone formation assay

Cell suspensions were prepared, and each group of cells was inoculated in 6-well plates with 500~1,000 cells per well, respectively, and 2 mL of complete medium was added, and cultured for about 1 week until clone spheres visible to the naked eye were produced. After methanol fixation for 30 min, the cells were stained with Giemsa's stain, rinsed and air-dried, photographed and the number of clone formation was calculated for each group.

2.13 Statistical analysis

The data analysis was conducted using GraphPad Prism 6.0 software (San Diego, CA, USA) and presented as mean ± S.D. each experiment had three replicates (n=3). For comparisons among multiple groups, we utilized one-way analysis of variance (ANOVA) followed by Tukey's multiple comparison posttest to assess statistical significance. Additionally, Student's unpaired t-test was employed to compare differences between two groups. Statistical significance was defined at $P < 0.05$.

Results

In vitro assay verifies that KAT2A regulates asthma through ~~iron death~~ferroptosis

We constructed the OVA asthma model, and through qPCR experiment and protein immunoblotting experiment, we found that the protein in the OVA model group was low expression, and the protein in the KAT2A group was high expression and significantly higher than that in the blank control group ($p<0.01$) (Figure 1A and B). ~~Then the lung tissue was stained,~~ Masson staining results of the lung tissue showed that the blank group bronchial tissue structure is normal, no airway wall and smooth muscle thickening, no obvious inflammatory cell infiltration (Figure 1C); OVA asthma model group can be seen around the inflammatory cell infiltration is obvious, the blue collagen deposition around the tracheal blood vessels further aggravated, epithelial cell fibrosis; OVA + control group have different degrees of pathology changes. In the OVA+control group, the pathological changes were reduced to different degrees, with a small amount of inflammatory cell infiltration around the airways, reduced thickness of the basement membrane and smooth muscle, and reduced collagen deposition; in the OVA+KAT2A group, the tissue results returned to normal, with no obvious inflammatory cell infiltration, and TUNEL staining showed that the nuclei of the cells in the control group were blue, while the nuclei of apoptotic cells were stained in brownish-yellow in the OVA asthma model group, with a significant increase in the number of positive cells; the number of positive cells was significantly reduced in the OVA+KAT2A group (Figure 1D). HE staining showed that in the blank group, the bronchial mucosa was smooth and intact, the lumen was smooth, the alveolar structure was intact, there was no thickening of the smooth muscle layer and the wall, and there was no obvious inflammatory cell infiltration; in the OVA model group, a large number of inflammatory cell infiltration was seen in the lung tissues, with disorganized airway epithelial structure, edema of mucous membranes, obvious narrowing of lumen, and a large number of inflammatory cells exuding from the peripheral part of the bronchial tubes; in the OVA+KAT2A group, the nuclei of the bronchial mucosa were blue, and the nuclei of apoptotic cells were stained in brownish-yellow, with a significantly greater number of positive cells. KAT2A group bronchial mucosal congestion and edema was reduced, inflammatory cells were reduced, the basement membrane was mildly hyperplastic and hypertrophic, and epithelial cell shedding was reduced (Figure 1E). In order to investigate the effect of KAT2A on inflammation, we counted macrophages, eosinophils, lymphocytes, and neutrophil cells, and found that the number of BALF, macrophages, eosinophils, lymphocytes, and neutrophils cells in the OVA asthma model group was significantly higher than that in the blank group ($p< 0.01$), and with the addition of KAT2A, the number of these cells decreased, especially the number of cells of eosinophils, lymphocytes, and neutrophils decreased significantly ($p<0.01$) (Figure 1F). Next, inflammatory factors were detected by qPCR and verified by ELISA, and it was found that inflammatory factors were significantly highly expressed in the OVA asthma model group ($p<0.01$), and with the addition of KAT2A, the expression of inflammatory factors was significantly reduced ($p<0.01$) (Figure 1G and H). Changes in the levels of Fe2+, Iron, lipid ROS, MDA, and SOD in lung tissues were observed using biochemical assays, and the results showed that the expression was significantly higher in the model group compared with the control group ($p<0.01$), and significantly lower with the addition of KAT2A ($p<0.01$) (Figure 1I). Detection of GPX4 and SLC7A11 levels in lung tissues using Western botting showed a significant decrease ($p<0.01$) in the model group, and again KAT2A reversed this result (Figure 1J).

In ~~vivo~~vitro assay to validate KAT2A-mediated histone succinylation modification and its

effect on asthma

The viability of HBE cells was detected by CCK-8 and cell cloning assay, which was firstly categorized into four groups: control, IL-13, IL-13+control and IL-13 +KAT2A, and it was found that IL-13 caused a significant decrease in cell viability, and KAT2A reversed this result (Figure 2 A and B). KAT2A was then analyzed by qPCR assay (Figure 2C) and protein immunoblotting assay (Figure 2D), and as with the previous results, protein expression was significantly reduced in the IL-13 group ($p<0.01$) and increased in the IL-13 +KAT2A group ($p<0.01$). Then we used biochemical assays to observe the changes in the levels of $Fe2+$, Iron, lipid ROS, MDA, and SOD, and found that IL-13 significantly elevated the levels of $Fe2+$, Iron, lipid ROS, MDA, and SOD ($p<0.01$), and KAT2A reversed this result (Figure 2E). Finally, GPX4 and SLC7A11 levels were detected using Western botting, and it was found that the levels of GPX4 and SLC7A11 were significantly decreased in the IL-13 group ($p<0.01$), and significantly increased in the IL-13 + KAT2A group ($p<0.01$) (Figure 2F).

KAT2A regulates asthma through SLC7A11

SLC7A11, as an important component of the Xc-system, a classical pathway of ~~iron death~~ferroptosis, can effectively regulate the process of cellular ~~iron death~~ferroptosis. In order to study how KAT2A regulates asthma through SLC7A11, we analyzed SLC7A11 by ChIP-qPCR, and found that SLC7A11 was significantly increased in the KAT2A group ($p<0.01$) (Figure 3A), and then we analyzed the expression of H3K79succ and RNA pol II on the promoter of SLC7A11, as shown in the figure, KAT2A significantly increased H3K79succ and RNA pol II expression ($p<0.01$) (Figure 3B and C). To further investigate the relationship between KAT2A and SLC7A11, SLC7A11 was analyzed by qPCR and immunoblotting assay, which showed that SLC7A11 expression was significantly increased in the KAT2A group ($p<0.01$) (Figure 3D and E). So we transfected with siKAT2A-1 and siKAT2A-2 and analyzed KAT2A and SLC7A11 by qPCR and WB, and the results showed that silencing KAT2A significantly decreased the expression of SLC7A11 ($p<0.01$) (Figure 3F and G). This suggests that KAT2A controls ~~iron death~~ferroptosis and subsequently regulates asthma through SLC7A11.

KAT2A-mediated histone succinylation modification is involved in asthma by inhibiting ~~iron death~~ferroptosis

To further validate that KAT2A regulates asthma by inhibiting ferroptosis, we added the ferroptosis inducer Erastin and observed whether the protective effect of KAT2A would be reversed. ~~To~~We investigate the effect of Erastin on HBE cell viability, we analyzed cell viability using CCK-8 and cell clone formation assay, and the results showed that KAT2A significantly increased cell viability ($p<0.01$), which was directly reversed by Erastin (Figure 4A and B). We then analyzed $Fe2+$, Iron, lipid ROS, MDA, and SOD, and found that Erastin significantly increased the levels of $Fe2+$, Iron, lipid ROS, MDA, and SOD ($p<0.01$) (Figure 4C).WB results showed that Erastin significantly decreased the expression of GPX4 and SLC7A11 ($p<0.01$) (Figure 4D). We stained the lung tissues of OVA asthma model, Masson staining results showed that Erastin caused significant inflammatory cell infiltration in lung tissues, further aggravation of blue collagen deposition around the tracheal blood vessels, and fibrosis of epithelial cells (Figure 4E). TUNEL staining results showed that Erastin stained nuclei of apoptotic cells brownish-yellow color, and the number of positive cells was significantly increased (Figure 4F). HE staining results showed that t~~T~~he results of HE staining showed that the lung tissue of OVA+KAT2A+Erastin group was infiltrated with a large number of inflammatory cells, the airway epithelial structure

was disorganized, the mucous membrane was edematous, the lumen was obviously narrowed, and there was a large number of inflammatory cells exuding from the peribronchial area (Figure 4G). In order to investigate whether Erastin could improve the inflammatory response in asthma, we counted macrophages, eosinophils, lymphocytes, and neutrophilic leukocytes cells, and found that Erastin significantly increased the number of immune cells ($p<0.01$) (Figure 4H). Next, inflammatory factors were detected by qPCR and verified by ELISA, and with the addition of KAT2A, the expression of inflammatory factors was significantly reduced ($p<0.01$), a result that was directly reversed after the addition of Erastin (Figure 4I and J). Finally, we used biochemical assays to observe the changes in the levels of Fe2+, Iron, lipid ROS, MDA, and SOD in lung tissues, and found that Erastin significantly increased their levels while significantly reducing the expression of GPX4 and SLC7A11 ($p<0.01$) (Figure 4K and L).

Discussion

Asthma is a chronic inflammatory airway disease with recurrent episodes of wheezing, shortness of breath, chest tightness or cough as the main clinical manifestations [21]. Due to urbanization and lifestyle changes, the prevalence of asthma is on the rise year by year. Asthma not only seriously affects the quality of life of patients, but also occupies a large amount of medical resources and increases the socio-economic burden, therefore, effective prevention and treatment of asthma remains an important clinical task and challenge. Some studies have found that iron death is involved in asthma [22], and others have found that the acetylation of KAT2A is involved in the regulation of inflammation, oxidative stress, and lipids [23], so we we explored the role of KAT2A-mediated histone succinimidylation modification involved in asthma through the inhibition of iron death, and determined the mechanism of its function.

In exploring the mechanisms of asthma treatment, animal models are often needed to explore the pathogenesis and therapeutic mechanisms of asthma. The most commonly used animals are BALB/C, C57BL/6J mice and rats, and the most commonly used modeling reagent is ovalbumin (OVA), which has strong immunogenicity and is an ideal allergen [24]. Ovalbumin promotes OVA-specific production of Il-4, Il-5, IL-10, and IL-13 by Th2 cells and production of antigen-specific IgE and IgG[25]. In this study we constructed the OVA asthma model to verify that KAT2A regulates asthma through iron death. As a complex and heterogeneous disease due to gene-environment interactions, the main features of asthma include chronic inflammation of the airways and airway remodeling [26]. Among them, airway remodeling is the main cause of progressive lung function decline and irreversible airflow limitation in asthma patients, and its pathological changes include subepithelial fibrosis, airway smooth muscle hypertrophy and hyperplasia, collagen deposition, and angiogenesis [27]. In the present study we found that KAT2A reduced inflammatory cell infiltration, led to a decrease in basement membrane and smooth muscle thickness, and reduced collagen deposition. Erastin, a small molecule antitumor drug also the first iron death inhibitor discovered can cause significant inflammatory cell infiltration in lung tissue, further aggravation of blue collagen deposition around airway vessels, and epithelial cell fibrosis. Asthma is a chronic inflammatory respiratory disease of the airways mediated by a variety of inflammatory cells and cellular components such as BALF, macrophages, eosinophils, lymphocytes, and neutrophils [28]. Airway inflammatory, airway hyperresponsiveness and widespread, reversible airflow obstruction often accompany their presence [29]. So we tested these cells and found that KAT2A reduced the number of these cells and Erastin reversed this result.

Formatted: Font color: Accent 6, Highlight

Meanwhile interleukin-13 (IL-13), a helper T cell 2-specific cytokine, is a pleiotropic regulator widely involved in inflammatory and immune diseases, and is important in the pathogenesis of asthma patients [30]. We verified that KAT2A mediates succinylation modification of IL-13 and participates in the development of asthma by in vivo assays.

The antioxidant system is an important defense system during the occurrence of iron death.SystemXc-system is a cystine/glutamate reverse transporter on the cell membrane, which is a heterodimer composed of SLC7A11 and SLC3A2 linked by disulfide bonds [31].SLC7A11, as an important part of the classical pathway of iron death, the Xc-system, is able to effectively regulate the process of cellular iron death[32]. In System Xc-system, glutathione peroxidase 4 (GPX4) inhibits the hydroperoxidation of lipids such as phospholipids and cholesterol, and synergizes with GSH to reduce the accumulation of lipid hydroperoxides, thus inhibiting the occurrence of iron death [33]. It has been shown that low maternal blood selenium concentration leads to reduced GPX4 activity in fetal airway epithelium and impaired antioxidant defense against oxidative stress, resulting in epithelial damage, which is involved in the development of asthma[34]. In order to investigate how KAT2A regulates asthma through SLC7A11, we analyzed it by ChIP-qPCR, and found that KAT2A significantly increased the expression of promoter H3K79succ and RNA pol II of SLC7A11, which suggests that KAT2A controls iron death and then regulates asthma through SLC7A11.

There are multiple pathways to control iron death, and damage-associated molecular patterns (DAMPS) is an important pathway for exerting biological effects of iron death , DAMPS is a general term for endogenous danger signals released by the body after injury, including high-mobility group egg self B1 (HMGB1), interleukin-1β (-1β), tumor necrosis factor-a (TNF-a), vascular endothelial growth factor (VEGF), and interleukin 33 (IL-33) [35]. Therefore we tested these inflammatory factors and found that KAT2A significantly reduced their levels, and again Erastin reversed this result. In addition iron death is a mode of cell death caused by lipid peroxidation that leads to the destruction of cell membrane structure, and its main features include mitochondrial morphology crumpling, increased ROS levels, GSH depletion, abnormal iron metabolism, and accumulation of lipid peroxidation products [36][37]. We analyzed Fe2+, Iron, lipid ROS, MDA, and SOD and found that KAT2A reduced their levels, and Erastin significantly increased their levels while significantly reducing the expression of GPX4 and SLC7A11.

Asthma is a chronic inflammatory airway disease characterized by recurrent wheezing, shortness of breath, chest tightness, or coughing[15]. Due to urbanization and changes in lifestyle, the prevalence of asthma has been increasing yearly. It not only severely affects patients' quality of life but also consumes significant healthcare resources, exacerbating the socioeconomic burden. Therefore, effective prevention and treatment of asthma remain crucial clinical tasks and challenges. Previous studies have found that ferroptosis is involved in the pathogenesis of asthma, and the acetylation modification of KAT2A is associated with the regulation of inflammation, oxidative stress, and lipid metabolism[16, 17]. In this study, we delved into the role and mechanism of KAT2A-mediated histone succinylation in asthma progression by inhibiting ferroptosis.

In exploring asthma treatment mechanisms, animal models are often used to investigate the pathophysiology and therapeutic approaches of asthma[18]. This study constructed an OVA-induced asthma model to verify the role of KAT2A in regulating asthma through ferroptosis. Asthma, a complex and heterogeneous disease arising from gene-environment interactions, is primarily characterized by chronic airway inflammation and airway remodeling. Airway

Formatted: Font: (Default) Times New Roman

Formatted: Font: (Default) Times New Roman

Formatted: Indent: First line: 2 ch

Formatted: Font: (Default) Times New Roman

remodeling, the main cause of progressive lung function decline and irreversible airflow limitation in asthma patients, involves pathological changes such as subepithelial fibrosis, airway smooth muscle hypertrophy and hyperplasia, collagen deposition, and angiogenesis[19, 20]. In our study, we observed that KAT2A reduced inflammatory cell infiltration, decreased basement membrane and smooth muscle thickness, and diminished collagen deposition. Conversely, Erastin, a small-molecule antitumor drug and the first discovered ferroptosis inducer, led to significant inflammatory cell infiltration in lung tissue, aggravated blue collagen deposition around airway blood vessels, and induced epithelial cell fibrosis[14, 21]. Asthma is a chronic inflammatory respiratory disease mediated by various inflammatory cells and components (such as BALF, macrophages, eosinophils, lymphocytes, and neutrophils), often accompanied by airway inflammation, airway hyperreactivity, and reversible airflow obstruction[18]. Our analysis revealed that KAT2A decreased the number of these cells, while Erastin reversed this effect. Additionally, interleukin-13 (IL-13), a pleiotropic regulatory factor specific to T helper type 2 cells, plays a pivotal role in the pathogenesis of asthma by widely participating in inflammatory and immune diseases. Through in vivo experiments, we verified that KAT2A mediates the succinylation of IL-13 and is involved in asthma development.

Regarding how KAT2A-mediated succinylation directly regulates H3K79succ on the SLC7A11 promoter, although current research has shown that KAT2A significantly increases the expression of H3K79succ and RNA polymerase II on the SLC7A11 promoter, the specific molecular mechanism remains incompletely understood[22, 23]. It is hypothesized that KAT2A, as a histone-modifying enzyme, may directly bind to the SLC7A11 promoter region, transferring a succinyl group to the lysine 79 site (H3K79) of histone H3. This modification could alter the structure and function of chromatin, shifting it from a tight transcriptionally repressed state to an open and transcriptionally active state. Consequently, this promotes the binding of RNA polymerase II to the promoter, enhancing SLC7A11 transcription. ~~To illustrate this process more clearly, we have constructed the following model diagram (assuming the inclusion of a simple schematic showing the interaction between KAT2A, the SLC7A11 promoter, H3K79succ, and RNA polymerase II): KAT2A is represented by a circle, the SLC7A11 promoter by a straight line, the H3K79succ modification site by small protrusions on the promoter, and RNA polymerase II by a larger irregular shape. When KAT2A binds near the SLC7A11 promoter, it transfers the succinyl group to the H3K79 site, facilitating the binding of RNA polymerase II to the promoter and initiating the transcription process of SLC7A11. This model aids in intuitively understanding the potential mechanism of KAT2A-mediated succinylation in regulating SLC7A11 expression, although further research is needed for validation.~~

The antioxidant system is a crucial defensive mechanism during ferroptosis. The System Xc-, a cystine/glutamate antiporter on the cell membrane, consists of SLC7A11 and SLC3A2 connected by a disulfide bond to form a heterodimer[24]. SLC7A11, a vital component of the ferroptosis classic pathway Xc- system, effectively regulates the process of ferroptosis. Within the System Xc-, glutathione peroxidase 4 (GPX4) inhibits the hydroperoxidation of lipids such as phospholipids and cholesterol, and collaborates with GSH to reduce the accumulation of lipid hydroperoxides, thereby suppressing ferroptosis[25]. Studies have indicated that low maternal blood selenium concentrations can lead to decreased GPX4 activity in fetal airway epithelial cells, impairing antioxidant defense capabilities and contributing to epithelial damage involved in

Formatted: Font: (Default) Times New Roman

Formatted: Font: (Default) Times New Roman

Formatted: Font: (Default) Times New Roman

Formatted: Font: (Default) Times New Roman

Formatted: Indent: First line: 2 ch

Formatted: Indent: First line: 2 ch

Formatted: Font: (Default) Times New Roman

Formatted: Font: (Default) Times New Roman

asthma development[26]. To investigate how KAT2A regulates asthma through SLC7A11, we employed ChIP-qPCR analysis and found that KAT2A significantly increased the expression of H3K79succ and RNA polymerase II on the SLC7A11 promoter. This suggests that KAT2A controls ferroptosis through modulation of SLC7A11, thereby regulating asthma.

Ferroptosis regulation occurs through multiple pathways, and damage-associated molecular patterns (DAMPs) represent one of the significant routes for ferroptosis to exert its biological effects. DAMPs are endogenous danger signals released by the body upon injury, including high-mobility group protein B1 (HMGB1), interleukin-1β (IL-1β), tumor necrosis factor α (TNF-α), vascular endothelial growth factor (VEGF), and interleukin-33 (IL-33)[27]. Our examination of these inflammatory factors revealed that KAT2A significantly reduced their levels, while Erastin reversed this outcome. Furthermore, ferroptosis is a form of cell death characterized by the destruction of cell membrane structure due to lipid peroxidation. Its primary features include mitochondrial morphological shrinkage, elevated ROS levels, GSH depletion, abnormal iron metabolism, and the accumulation of lipid peroxidation products[7]. Our analysis of indicators such as Fe^{2+}, iron ions, lipid ROS, MDA, and SOD demonstrated that KAT2A lowered their levels, whereas Erastin significantly elevated these markers and concomitantly reduced the expression of GPX4 and SLC7A11.

From a clinical perspective, current asthma treatments primarily focus on anti-inflammatory and bronchodilator approaches[28]. However, the therapeutic effectiveness remains unsatisfactory for some patients, emphasizing the importance of identifying new therapeutic targets. Our study reveals that KAT2A-mediated histone succinylation is involved in asthma progression by inhibiting ferroptosis, offering a potential new target for asthma treatment. Compared to existing anti-IL-13 biologics, which alleviate asthma inflammation by blocking the IL-13 signaling pathway but can only intervene in a single cytokine and may cause drug resistance or adverse reactions in some patients, targeting KAT2A could regulate asthma pathogenesis at a more upstream epigenetic level. This approach not only modulates IL-13-related inflammatory responses but also impacts multiple pathological aspects of asthma, such as reducing airway epithelial cell damage and improving airway remodeling, by inhibiting ferroptosis[29, 30]. Additionally, as an enzyme, KAT2A has the potential to be targeted by small molecule inhibitors or activators. Compared to biologics, small molecule drugs offer better stability, lower production costs, and more convenient administration routes. However, it's important to note that this discovery is still in the basic research stage, and further studies are needed to translate it into clinical applications. This includes validating KAT2A's role in larger clinical samples, developing specific drugs targeting KAT2A, and evaluating their safety and effectiveness. Nonetheless, our study opens up new avenues for asthma treatment with potential clinical translational value.

In conclusion, this study provides initial insights into the mechanistic role of KAT2A in asthma. However, further investigation is warranted to address various unanswered questions and ultimately contribute to more effective clinical strategies for asthma management.

Funding:

Authors acknowledge the following funders.

1. Qilu Health and health leading talent project（2021-1-6）; 2. Shandong Province traditional Chinese medicine science and technology development program （2013ZDZK-099）3. Natural science Foundation of Shandong Province （ZR2019MH108）

Author contribution:

J.H.: Conceptualization, Validation, Funding acquisition
H. D.: Formal analysis, Writing - Original Draft
S.L.: Visualization, Writing - Original Draft
J.L.: Methodology, Supervision, Writing - Review & Editing

Formatted: Font: (Default) Times New Roman, 10.5 pt
Formatted: Font: (Default) Times New Roman, 10.5 pt
Formatted: Font: (Default) Times New Roman, 10.5 pt
Formatted: Font: (Default) Times New Roman, 10.5 pt
Formatted: Font: (Default) Times New Roman, 10.5 pt
Formatted: Font: (Default) Times New Roman, 10.5 pt
Formatted: Font: (Default) Times New Roman, 10.5 pt
Formatted: Font: (Default) Times New Roman, 10.5 pt
Formatted: Font: (Default) Times New Roman, 10.5 pt
Formatted: Font: (Default) Times New Roman, 10.5 pt
Formatted: Font: (Default) Times New Roman, 10.5 pt
Formatted: Font: (Default) Times New Roman, 10.5 pt
Formatted: Font: Bold
Formatted: Font: Not Bold

Figure legends

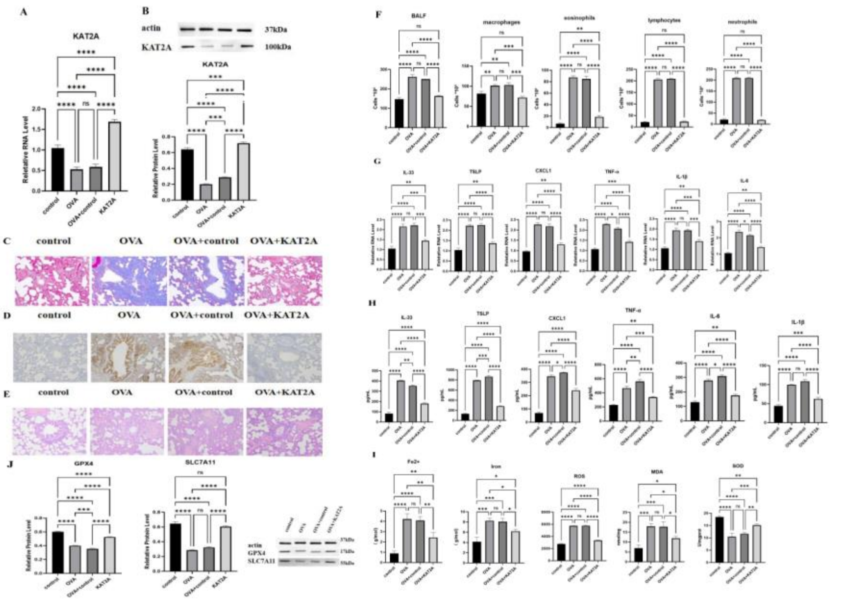

Figure 1. In ~~vitro~~vivo assay verifies that KAT2A regulates asthma through ~~iron death~~ferroptosis (A-J), qPCR analysis of KAT2A in C57BL/6J (A), WB analysis of KAT2A in C57BL/6J (B), Masson staining of C57BL/6J (C), TUNEL staining of C57BL/6J (D), –H&E staining of C57BL/6J (E), Immuno Cytometrics including the cells level of BALF, macrophages, eosinophols, lymphocytes, neutrophils in C57BL/6J (F), qPCR analysis of inflammatory factors in C57BL/6J (G), ELISA analysis of inflammatory factors in C57BL/6J (H), Fe2+, Iron, lipid ROS, MDA, SOD analysis (I), WB analysis of GPX4 and SLC7A11 in C57BL/6J (J). ****P>0.0001, ***P>0.001, **P<0.01, *P<0.05, ns: not significant.

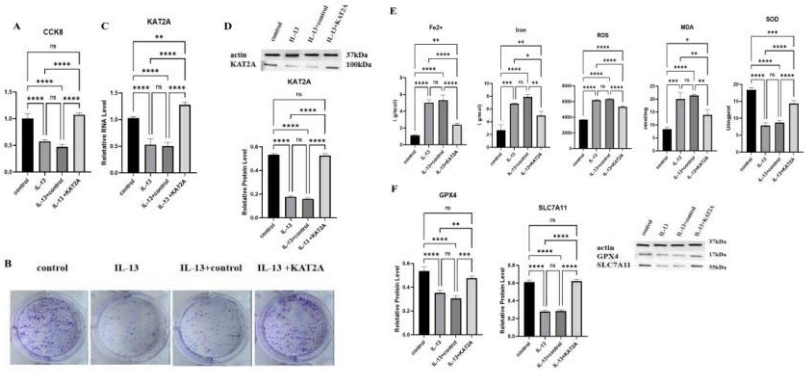

Figure 2. In vitro~~vivo~~ assay to validate KAT2A-mediated histone succinylation modification and its effect on asthma (A-F) CCK-8 analysis of cell proliferation (A), Clone formation analysis of

Formatted: Font: (Default) Times New Roman, 10.5 pt, Not Bold, Font color: Auto, Pattern: Clear

cell proliferation (B), qPCR analysis of KAT2A in HBE cells (C), WB analysis of KAT2A in HBE cells (D), Fe^{2+}, Iron, lipid ROS, MDA, SOD analysis in HBE cells (E), WB analysis of GPX4 and SLC7A11 in HBE cells (F). ****P>0.0001, ***P>0.001, **P<0.01, *P<0.05, ns: not significant. Each experiment had three replicates (n=3), the data analysis was conducted using GraphPad Prism 6.0 software (San Diego, CA, USA) and presented as mean ± S.D.and presented as mean ± S.D.

~~(F)~~

Formatted: Left, Indent: Left: 0", Hanging: 1 ch, First line: -1 ch

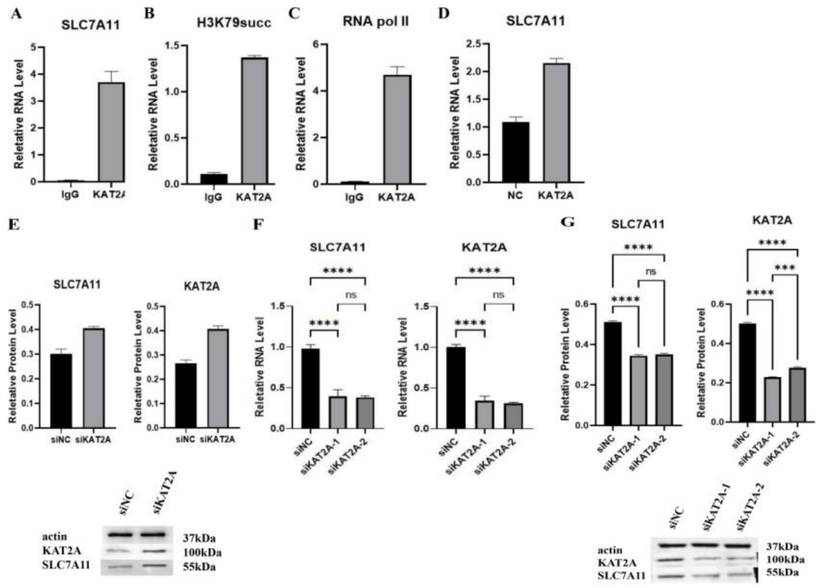

Figure 3. KAT2A regulates asthma through SLC7A11 (A-G) ChIP-qPCR analysis of SLC7A11 (A), ChIP-qPCR analysis of H3K79succ on the promoter of SLC7A11 (B), ChIP-qPCR analysis of the promoter of SLC7A11 on RNA pol II (C), qPCR analysis of SLC7A11 (D and F), WB analysis of KAT2A and SLC7A11 (E and G). ****P>0.0001, ***P>0.001, **P<0.01, *P<0.05, ns: not significant. Each experiment had three replicates (n=3), the data analysis was conducted using GraphPad Prism 6.0 software (San Diego, CA, USA) and presented as mean ± S.D.and presented as mean ± S.D.

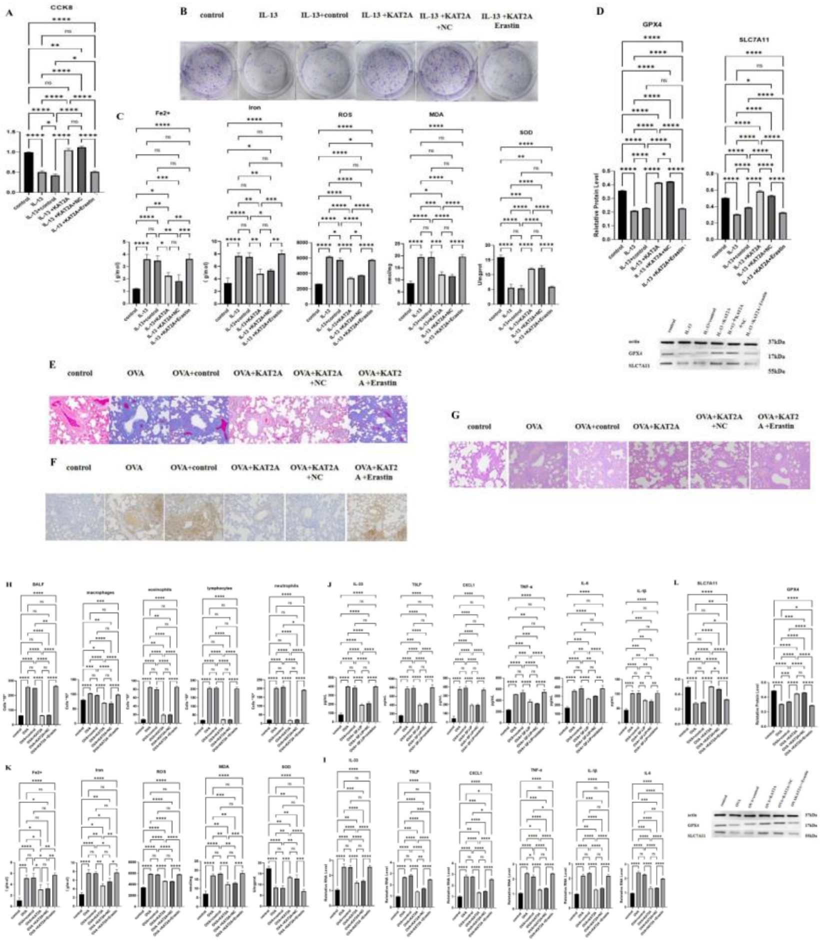

Figure 4. KAT2A-mediated histone succinylation modification is involved in asthma by inhibiting ~~iron death~~ferroptosis (A-L) CCK-8 analysis of cell proliferation (A), Clone formation analysis of cell proliferation (B), Fe2+, Iron, lipid ROS, MDA, and SOD analysis (C and K), WB analysis of GPX4 and SLC7A11 (D and L), Masson staining (E), TUNEL staining (F), H&E staining (G), Immunocytometrics (H), qPCR analysis of inflammatory factors (I), ELISA analysis of inflammatory factors (J).

~~[1] Jesenak M, Durdik P, Oppova D, et al. Dysfunctional mucociliary clearance in asthma and~~

airway remodeling - New insights into an old topic. Respir Med. 2023 Nov;218:107372.
[2] Patrick R, Hensher M, Suphioglu C, Huxley R. Asthma-The canary in the Australian coalmine: Making the links between climate change, fossil fuel and public health outcomes. Health Promot J Austr. 2024 Apr;35(2):340-344.
[3] Tsai JJ, Yen CY, Hsu CH, et al. Immunomodulatory effects of modified Liu-Wei-Di-Huang-Wan Traditional Chinese medicine on allergic asthmatic mice. Allergy Asthma Clin Immunol. 2023 Apr 26;19(1):35.
[4] Savin IA, Zenkova MA, Sen'kova AV. Bronchial Asthma, Airway Remodeling and Lung Fibrosis as Successive Steps of One Process. Int J Mol Sci. 2023 Nov 7;24(22):16042.
[5] Ramakrishnan RK, Al Heialy S, Hamid Q. Role of IL-17 in asthma pathogenesis and its implications for the clinic. Expert Rev Respir Med. 2019 Nov;13(11):1057-1068.
[6] Savin IA, Zenkova MA, Sen'kova AV. Bronchial Asthma, Airway Remodeling and Lung Fibrosis as Successive Steps of One Process. Int J Mol Sci. 2023 Nov 7;24(22):16042.
[7] Cha J, Choi S. Gene-Smoking Interaction Analysis for the Identification of Novel Asthma-Associated Genetic Factors. Int J Mol Sci. 2023 Jul 31;24(15):12266.
[8] Hwang H, Jang JH, Lee E, et al. Prediction of the number of asthma patients using environmental factors based on deep learning algorithms. Respir Res. 2023 Dec 1;24(1):302.
[9] Huang YF, Ou GC, Ma SH, et al. Effect of icariin on the H2O2-induced proliferation of mouse airway smooth muscle cells through miR-138-5p regulating SIRT1/AMPK/PGC-1α axis. Int J Immunopathol Pharmacol. 2023 Jan-Dec;37:3946320231151515.
[10] Lin X, Wang L, Lu X, et al. Targeting of G-protein coupled receptor 40 alleviates airway hyperresponsiveness through RhoA/ROCK1 signaling pathway in obese asthmatic mice. Respir Res. 2023 Feb 17;24(1):56.
[11] Ali MK, Kim RY, Brown AC,et al.Crucial role for lung iron level and regulation in the pathogenesis and severity of asthma. Eur Respir J. 2020 Apr 23;55(4):1901340.
[12] Anmangandla A, Ren Y, Fu Q, Zhang S, Lin H. The Acyl-CoA Specificity of Human Lysine Acetyltransferase KAT2A. Biochemistry. 2022 Sep 6;61(17):1874-1882.
[13] Li H, Li C, Yang LZ, Liu J. Integrative analysis of histone acetyltransferase KAT2A in human cancer. Cancer Biomark. 2023;38(4):443-463.
[14] Yu Z, Ding M, Cai Y,et al. Histone regulator KAT2A acts as a potential biomarker related to tumor microenvironment and prognosis of diffuse large B cell lymphoma. BMC Cancer. 2023 Oct 3;23(1):934.
[15] Zhou J, Yan X, Liu Y, Yang J. Succinylation of CTBP1 mediated by KAT2A suppresses its inhibitory activity on the transcription of CDH1 to promote the progression of prostate cancer. Biochem Biophys Res Commun. 2023 Apr 2;650:9-16
[16] Dong Z, He W, Lin G,et al. Histone acetyltransferase KAT2A modulates neural stem cell differentiation and proliferation by inducing degradation of the transcription factor PAX6. J Biol Chem. 2023 Mar;299(3):103020.
[17] Wang Y, Guo YR, Liu K,et al.KAT2A coupled with the α-KGDH complex acts as a histone H3 succinyltransferase. Nature. 2017 Dec 14;552(7684):273-277.
[18] Ye L, Yu Z, He L,et al.KAT2A-mediated succinylation modification of notch1 promotes the proliferation and differentiation of dental pulp stem cells by activating notch pathway. BMC Oral Health. 2024 Mar 31;24(1):407.
[19] Wiesel-Motiuk N, Assaraf YG. The key roles of the lysine acetyltransferases KAT6A and

KAT6B in physiology and pathology. Drug Resist Updat. 2020 Dec;53:100729.
[20] Zhang Y, Gao Y, Ding Y,et al. Targeting KAT2A inhibits inflammatory macrophage activation and rheumatoid arthritis through epigenetic and metabolic reprogramming. MedComm (2020). 2023 Jun 11;4(3):e306.
[21] Camoretti-Mercado B, Lockey RF. Airway smooth muscle pathophysiology in asthma. J Allergy Clin Immunol. 2021 Jun;147(6):1983-1995.
[22] Lv X, Dong M, Tang W,et al. Ferroptosis, novel therapeutics in asthma. Biomed Pharmacother. 2022 Sep;153:113516.
[23] Zhang Y, Gao Y, Ding Y,et al. Targeting KAT2A inhibits inflammatory macrophage activation and rheumatoid arthritis through epigenetic and metabolic reprogramming. MedComm (2020). 2023 Jun 11;4(3):e306.
[24] Liang S, Zhao Y, Chen G, Wang C. Isoorientin ameliorates OVA-induced asthma in a murine model of asthma. Exp Biol Med (Maywood). 2022 Aug;247(16):1479-1488.
[25] Majewska-Szczepanik M, Askenase PW, Lobo FM,et al.Epicutaneous immunization with ovalbumin and CpG induces TH1/TH17 cytokines, which regulate IgE and IgG2a production. J Allergy Clin Immunol. 2016 Jul;138(1):262-273.e6.
[26] Zhang Y, Saradna A, Ratan R,et al.RhoA/Rho-kinases in asthma: from pathogenesis to therapeutic targets. Clin Transl Immunology. 2020 Apr 29;9(5):e01134.
[27] Joseph C, Tatler AL. Pathobiology of Airway Remodeling in Asthma: The Emerging Role of Integrins. J Asthma Allergy. 2022 May 11;15:595-610.
[28] León B, Ballesteros-Tato A. Modulating Th2 Cell Immunity for the Treatment of Asthma. Front Immunol. 2021 Feb 10;12:637948.
[29] Wang L, Zhou L, Zheng P,et al. Mild asthma is not mild: risk factors and predictive biomarkers for severe acute exacerbations and progression in mild asthma. Expert Rev Respir Med. 2023 Dec;17(12):1261-1271.
[30] Moran A, Pavord ID. Anti-IL-4/IL-13 for the treatment of asthma: the story so far. Expert Opin Biol Ther. 2020 Mar;20(3):283-294.
[31] Zhang C, Shafaq-Zadah M, Pawling J,et al. SLC3A2 N-glycosylation and Golgi remodeling regulate SLC7A amino acid exchangers and stress mitigation. J Biol Chem. 2023 Dec;299(12):105416.
[32] Bi R, Hu R, Jiang L,et al. Butyrate enhances erastin-induced ferroptosis of lung cancer cells via modulating the ATF3/SLC7A11 pathway. Environ Toxicol. 2024 Feb;39(2):529-538.
[33] Bao L, Jin Y, Han J,et al. Berberine Regulates GPX4 to Inhibit Ferroptosis of Islet β Cells. Planta Med. 2023 Mar;89(3):254-261.
[34] Samo SP, Malhi M, Kachiwal AB,et al. Supranutritional selenium level minimizes high concentrate diet-induced epithelial injury by alleviating oxidative stress and apoptosis in colon of goat. BMC Vet Res. 2020 Nov 27;16(1):462.
[35] Lei H, Li Q, Pei Z,et al. Nonferrous Ferroptosis Inducer Manganese Molybdate Nanoparticles to Enhance Tumor Immunotherapy. Small. 2023 Nov;19(45):e2303438.
[36] Itagaki K, Riça I, Konecna B,et al. Role of Mitochondria-Derived Danger Signals Released After Injury in Systemic Inflammation and Sepsis. Antioxid Redox Signal. 2021 Nov 20;35(15):1273-1290.
[37] Kwun MS, Lee DG. Ferroptosis-Like Death in Microorganisms: A Novel Programmed Cell Death Following Lipid Peroxidation. J Microbiol Biotechnol. 2023 Aug 28;33(8):992-997.

References

[1] Jesenak M, Durdik P, Oppova D, et al. Dysfunctional mucociliary clearance in asthma and airway remodeling - New insights into an old topic. Respir Med. 2023. 218: 107372.

[2] Patrick R, Hensher M, Suphioglu C, Huxley R. Asthma-The canary in the Australian coalmine: Making the links between climate change, fossil fuel and public health outcomes. Health Promot J Austr. 2024. 35(2): 340-344.

[3] Tsai JJ, Yen CY, Hsu CH, Yu SJ, Chen CH, Liao EC. Immunomodulatory effects of modified Liu-Wei-Di-Huang-Wan Traditional Chinese medicine on allergic asthmatic mice. Allergy Asthma Clin Immunol. 2023. 19(1): 35.

[4] Savin IA, Zenkova MA, Sen'kova AV. Bronchial Asthma, Airway Remodeling and Lung Fibrosis as Successive Steps of One Process. Int J Mol Sci. 2023. 24(22): 16042.

[5] Zhao J, Dar HH, Deng Y, et al. PEBP1 acts as a rheostat between prosurvival autophagy and ferroptotic death in asthmatic epithelial cells. Proc Natl Acad Sci U S A. 2020. 117(25): 14376-14385.

[6] Yamada K, St Croix C, Stolz DB, et al. Compartmentalized mitochondrial ferroptosis converges with optineurin-mediated mitophagy to impact airway epithelial cell phenotypes and asthma outcomes. Nat Commun. 2024. 15(1): 5818.

[7] Jiang X, Stockwell BR, Conrad M. Ferroptosis: mechanisms, biology and role in disease. Nat Rev Mol Cell Biol. 2021. 22(4): 266-282.

[8] Pan L, He B, Han Y, Yuan D, Duan X, Wang Y. Yanghe Pingchuan granules induce ferroptosis in airway smooth muscle cells to improve bronchial asthma via the METTL3/P53/SLC7A11 signaling pathway. Phytomedicine. 2025. 139: 156480.

[9] Dai Y, Cui C, Jiao D, Zhu X. JAK/STAT signaling as a key regulator of ferroptosis: mechanisms and therapeutic potentials in cancer and diseases. Cancer Cell Int. 2025. 25(1): 83.

[10] Wang Y, Guo YR, Liu K, et al. KAT2A coupled with the α-KGDH complex acts as a histone H3 succinyltransferase. Nature. 2017. 552(7684): 273-277.

[11] Wang W, Chen X, Wei W. TRIM22 mechanism promoting KAT2A ubiquitination degradation to regulate ferroptosis in hepatocellular carcinoma cell invasion and metastasis. Histol Histopathol. 2024 : 18856.

[12] Conde E, Bertrand R, Balbino B, et al. Dual vaccination against IL-4 and IL-13 protects against chronic allergic asthma in mice. Nat Commun. 2021. 12(1): 2574.

[13] Berry M, Brightling C, Pavord I, Wardlaw A. TNF-alpha in asthma. Curr Opin Pharmacol. 2007. 7(3): 279-82.

[14] Zhang Y, Tan H, Daniels JD, et al. Imidazole Ketone Erastin Induces Ferroptosis and Slows Tumor Growth in a Mouse Lymphoma Model. Cell Chem Biol. 2019. 26(5): 623-633.e9.

[15] Miller RL, Grayson MH, Strothman K. Advances in asthma: New understandings of asthma's natural history, risk factors, underlying mechanisms, and clinical management. J Allergy Clin Immunol. 2021. 148(6): 1430-1441.

[16] Zhang Y, Gao Y, Ding Y, et al. Targeting KAT2A inhibits inflammatory macrophage activation and rheumatoid arthritis through epigenetic and metabolic reprogramming. MedComm (2020). 2023. 4(3): e306.

[17] Liu Y, Zeng JM, Zhao H, et al. Mechanism of KAT2A regulation of H3K36ac in manganese-induced oxidative damage to mitochondria in the nervous system and intervention by curcumin. Ecotoxicol Environ

Saf. 2024. 273: 116155.
[18] Woodrow JS, Sheats MK, Cooper B, Bayless R. Asthma: The Use of Animal Models and Their Translational Utility. Cells. 2023. 12(7): 1091.
[19] Banno A, Reddy AT, Lakshmi SP, Reddy RC. Bidirectional interaction of airway epithelial remodeling and inflammation in asthma. Clin Sci (Lond). 2020. 134(9): 1063-1079.
[20] Varricchi G, Ferri S, Pepys J, et al. Biologics and airway remodeling in severe asthma. Allergy. 2022. 77(12): 3538-3552.
[21] Li C, Chen F, Lin L, Li J, Zheng Y, Chen Q. CSE triggers ferroptosis via SIRT4-mediated GNPAT deacetylation in the pathogenesis of COPD. Respir Res. 2023. 24(1): 301.
[22] Qin YP, Yu HB, Yuan SY, et al. KAT2A Promotes Hepatitis B Virus Transcription and Replication Through Epigenetic Regulation of cccDNA Minichromosome. Front Microbiol. 2021. 12: 795388.
[23] KAT2A Is a α-KGDH-Dependent Histone Succinyltransferase. Cancer Discov. 2018. 8(2): 138.
[24] Wang L, Liu Y, Du T, et al. ATF3 promotes erastin-induced ferroptosis by suppressing system Xc(). Cell Death Differ. 2020. 27(2): 662-675.
[25] Chen X, Yu C, Kang R, Kroemer G, Tang D. Cellular degradation systems in ferroptosis. Cell Death Differ. 2021. 28(4): 1135-1148.
[26] Shaheen SO, Rutterford CM, Lewis SJ, et al. Maternal selenium status in pregnancy, offspring glutathione peroxidase 4 genotype, and childhood asthma. J Allergy Clin Immunol. 2015. 135(4): 1083-1085.e3.
[27] Murao A, Aziz M, Wang H, Brenner M, Wang P. Release mechanisms of major DAMPs. Apoptosis. 2021. 26(3-4): 152-162.
[28] Bruce P, Hatter L, Beasley R. Anti-inflammatory reliever therapy in asthma: The evidence mounts but more is needed. Respirology. 2020. 25(8): 776-778.
[29] Parulekar AD, Kao CC, Diamant Z, Hanania NA. Targeting the interleukin-4 and interleukin-13 pathways in severe asthma: current knowledge and future needs. Curr Opin Pulm Med. 2018. 24(1): 50-55.
[30] McCann MR, Kosloski MP, Xu C, Davis JD, Kamal MA. Dupilumab: Mechanism of action, clinical, and translational science. Clin Transl Sci. 2024. 17(8): e13899.

AUTHORSHIP RESPONSIBILITY AND COPYRIGHT TRANSFER

Manuscript Number:

Manuscript Title: Molecular mechanism of modified KAT2A-mediated histone succinylation in asthma through inhibition of iron death

Corresponding Author: Jun-feng Li

E-mail: lijunfeng0008@163.com

Mailing Address and telephone numbers: Pediatrics of traditional Chinese medicine, Qingdao Women and Children's Hospital, Qingdao, 266000, China.

All authors must read the following statements and sign (original ink signature) this form.

The undersigned authors transfer all copyright ownership of the manuscript to CLINICS. Manuscripts submitted to CLINICS may not be under simultaneous consideration by any other publication and should not have been published previously in similar form.
All authors must have contributed to the study and/or manuscript. All are responsible for the contents and warrant that he or she had a significant participation in the work and has reviewed the updated manuscript submitted for consideration.
Studies involving experimental animals must conform to the guiding principles of the Declaration of Helsinki. Human subjects must have given informed consent to the study as approved by the Committee on Ethics and Research at the author's institution.
The authors warrant that the manuscript is original. Neither this manuscript nor a similar one has been published nor shall be submitted for publication elsewhere while under consideration.
Authors warrant that data are used with the consent of the person generating the data
Authors understand that if the review of the submitted manuscript depends highly upon another manuscript submitted for publication but not yet published, a copy of that manuscript should be included with the submission.
The work does not infringe upon the statutory or common law copyright or any trademark registrations.
Previously published figures, tables or illustrations must be accompanied by written permission from the publishers to reproduce.
Any financial or other relations that could lead to a conflict of interest must be disclosed in the letter of submission.

Signature	Printed Name	Date
Han	Jian Han	15/2/2025
Du Hui-li	Hui-li Du	15/2/2025
Lu	Shan-shan Lu	15/2/2025
Li	Jun-feng Li	15/2/2025

Declaration of interests

☒ The authors declare that they have no known competing financial interests or personal relationships that could have appeared to influence the work reported in this paper.

☐The authors declare the following financial interests/personal relationships which may be considered as potential competing interests:
